# ZBTB1 Regulates Glycolysis and Chemotherapy Resistance Through Interacting with c-Myc in Triple Negative Breast Cancer

**DOI:** 10.3390/cancers18193156

**Published:** 2026-09-29

**Authors:** Na Qian, Songyao Sun, Cong Ouyang, Wenlong Xie, Shuhui Chen, Huizheng Xie, Songyu Li, Xinyu Wang, Yumeng Wang, Zihua Li, Lili Wang, Kuaiying Wu, Jiajun Cui

**Affiliations:** 1 The Center for Translational Medicine, Yichun University, Yichun 336000, China; 2 The Department of Biochemistry, School of Basic Medical Sciences, Yichun University, Yichun 336000, China; 3 Department of Radiation Oncology, Jiangxi Cancer Hospital, Nanchang 330029, China; 4 The Center for Applied Mathematics, School of Artificial Intelligence and Information Engineering, Yichun University, Yichun 336000, China

**Keywords:** TNBC, ZBTB1, SAP30L-AS1, c-Myc, glycolysis

## Abstract

**Simple Summary:**

Triple-negative breast cancer has few targeted treatment options and often develops resistance to chemotherapy. We studied the protein ZBTB1, which exists at low levels in this cancer and is linked to poorer patient survival. Our work explores how ZBTB1 controls tumour growth and chemotherapy response. We found that ZBTB1 binds to c-Myc to suppress glycolysis in Triple-negative breast cancer cells. This binding relies on a long non-coding RNA named SAP30L-AS1. When SAP30L-AS1 is lost in Adriamycin resistant cells, ZBTB1 is not able to interact with c-Myc. This change increases glycolysis and renders cancer cells resistant to Adriamycin. Our findings reveal a new pathway regulating chemotherapy resistance in tripe-negative breast cancer.

**Abstract:**

**Background/Objectives**: Our previous study identified ZBTB1 as a novel tumor suppressor in estrogen receptor-positive (ER+) breast cancer. Moreover, The Cancer Genome Atlas (TCGA) data analysis showed a much lower level of ZBTB1 in triple-negative breast cancer than in ER+ breast cancer. Triple negative breast cancer (TNBC) lacks effective targeted therapies and largely depends on chemotherapy, while chemotherapy resistance remains a major clinical challenge. This study aimed to explore the role of ZBTB1 in TNBC progression and chemotherapy resistance. **Methods**: We performed clinical survival analysis, in vitro and in vivo functional experiments, mass spectrometry screening, and mechanistic validation in TNBC and Adriamycin-resistant TNBC cells. **Results**: We found that ZBTB1 protein is downregulated in TNBC and this downregulation correlates with shorter patient survival. ZBTB1 regulates glycolysis and chemotherapy resistance in TNBC cells. Mechanistically, ZBTB1 interacts with c-Myc and suppresses its transcriptional activity in MDA-MB-231 and MDA-MB-453 but not in Adriamycin-resistant cells (MDA-MB-231/ADR and MDA-MB-453/ADR). LncRNA SAP30L-AS1 is required for maintaining the ZBTB1–c-Myc interaction in TNBC cells. SAP30L-AS1 is significantly depleted in Adriamycin-resistant cells, which disrupts the binding between ZBTB1 and c-Myc. Functional validation revealed that SAP30L-AS1 modulates glycolysis and chemotherapy resistance in TNBC cells in a ZBTB1-dependent manner. **Conclusions**: Collectively, our data indicate the key role of ZBTB1 in TNBC and ZBTB1 may serve as a novel approach for TNBC treatments.

## 1. Introduction

TNBC, defined by the lack of progesterone receptor (PR), estrogen receptor (ER) and human epidermal growth factor receptor 2 (HER2) amplification, has the highest recurrence rate and the worst outcome among all breast cancer subtypes [1,2,3,4,5]. TNBC accounts for about 15% of breast cancer cases. For lacking target therapy, TNBC treatment heavily relies on chemotherapy [6,7,8,9]. Unfortunately, after initial successful treatment TNBC tumors frequently acquire resistance to chemotherapy and the recurrence rates remain relatively high. Thus, chemotherapy resistance is one large obstacle for TNBC treatments [10,11,12].

Increasing evidence suggests that metabolic reprogramming plays a critical role in cancer [13,14,15,16]. The metabolic alterations in tumors were first discovered by Otto Warburg. He revealed that cancer cells prefer using glycolysis to generate ATP even when oxygen is sufficient and the phenomenon is termed as the Warburg effect. This effect promotes tumor growth in many types of cancer and revolutionized our understandings of cancer metabolism [17,18,19]. The respiratory impairment of cells is the prime cause of cancer and is responsible for the Warburg effect [20]. It has been shown that respiratory impairment and elevated glycolysis were observed in TNBC cells. Moreover, the enhanced glycolysis is believed to be linked with chemotherapy resistance in TNBC [20,21,22,23,24,25,26]. Key glycolytic enzymes, including lactate dehydrogenase A (LDHA) and hexokinase 2 (HK2), are frequently overexpressed in TNBC [27]. The inhibition of LDHA, the rate-limiting enzyme of glycolysis, suppressed tumor growth and overcame the chemo-resistance in TNBC [23,28]. Downregulation of HK2, the first enzyme of glycolysis, diminishes TNBC cell proliferation and tumor growth [29,30].

ZBTB1, a POK/ZBTB protein, was first defined as a determinant of lymphoid development [31]. An increasing number of studies suggest that ZBTB1 plays a critical role in replicating immune progenitors, allowing the generation of B-cell, T-cell and myeloid cells [32,33,34,35,36]. Our group first identified the role of ZBTB1 in cancer and we found that ZBTB1 functions as a novel tumor suppressor in ER^+^ breast cancer. ZBTB1 is downregulated in breast cancer and the downregulation indicates poor prognosis of patient survival [37]. Our data also demonstrate that ZBTB1 expression is significantly lower in TNBC than in ER^+^ breast cancer, yet its precise biological function remains to be fully elucidated.

In the present study, we observed that ZBTB1 protein expression is reduced in TNBC and the downregulation of ZBTB1 indicates a shorter survival in TNBC patients. Moreover, ZBTB1 suppresses glycolysis and chemotherapy resistance through interacting with c-Myc in TNBC cells.

## 2. Materials and Methods

### 2.1. Cell Culture and Plasmids

The human cell lines MDA-MB-231 (RRID:CVCL_0062), MDA-MB-436 (RRID:CVCL_0623), BT-549 (RRID:CVCL_1092) and MDA-MB-453 (RRID:CVCL_0418) were obtained from the Cell Bank of the Chinese Academy of Sciences (Shanghai, China). Cell lines were subjected to authenticating using STR profiling and mycoplasma detection (Supporting information). Cells were cultured in high-glucose DMEM (Dulbecco’s modified Eagle’s medium) supplemented with 10% fetal bovine serum (FBS, Gibco, São Paulo Brazil, Cat. No. 10270-106; multiple lots were used during experiments) and 1% penicillin-streptomycin antibiotics. Cells were incubated in a humidified incubator at 37 °C with 5% CO_2_. Adriamycin-resistant cells (MDA-MB-231/ADR and MDA-MB-453/ADR) were purchased from Shanghai Yansheng Industrial Co., Ltd. (Shanghai, China) and they were acquired by culturing parent cells with gradually increasing concentration of Adriamycin from 5 nM to 100 nM over a period of 1 year. The IC5o values of the Adriamycin-resistant cells were at least 5-fold higher than those of the corresponding parent cells. For maintaining the ADR-resistant phenotype, ADR-resistant cells were continuously cultured with 20 nM doxorubicin in medium. Before all experiments, cells were cultured in drug-free complete medium for 2 days to eliminate residual drug effects.

The reporter gene pMyc-TA-Luc was obtained from Beyotime (Shanghai, China). Full-length ZBTB1 was inserted into pCDH-CMV and SAP30L-AS1 was inserted into pLV3-CMV for lentivirus production in 293T cells according to our previous study [37] and the plasmid maps are presented in Figure S13. Sequences of shRNAs for ZBTB1 were as follows: shRNA#1: 5′-UGGCAAUGAACUACCUACAGCUAUA-3′ (491–515 of ZBTB1 mRNA); shRNA#2: 5′-GAGCCAAGUUCAACGGU-3′ (678–699 of ZBTB1 mRNA); Scramble shRNA: 5′-CGACCACATGAAGCAGCACGA-3′AATT-3′ [37].

### 2.2. Transient Transfections and Reporter Gene Assays

Cells were seeded into a 24-well plate and then transfected with plasmids as indicated using Lipofectamine3000 (Invitrogen, Carlsbad, CA, USA). Cells were seeded in 24-well plates, and 200 ng reporter plasmid together with 50 ng Renilla control plasmid was co-transfected with ZBTB1 1 μg per well. Empty vector plasmid was supplemented to balance total DNA among groups. After 24 h cells were collected to measure reporter gene activities. The reporter luciferase assay was conducted according to the instruction (Promega, Madison, WI, USA) as described previously [37]. At least three independent experiments were performed.

### 2.3. Lentivirus Packaging

Lentivirus packaging was performed in HEK293T cells. Briefly, the target overexpression or shRNA plasmid was co-transfected with the packaging plasmids psPAX2 and pMD2.G using Lipofectamine 3000 transfection reagent. The cell culture supernatant containing lentivirus was collected at 48 h and 72 h after transfection, followed by filtration through a 0.45 μm filter to remove cell debris. TNBC cells were infected with the prepared lentivirus. Puromycin (2 μg/mL) or G418 (500 μg/mL) screening was performed 48 h after transduction to generate stable cell pools. The transduction efficiency was routinely evaluated by observing fluorescent signals, and the average infection efficiency ranged from 70% to 80% in this study.

### 2.4. Western Blot

Western blot was conducted as described previously [37]. Total proteins were subjected to SDS-PAGE electrophoresis and transferred to PVDF membrane. The membrane was incubated with the antibodies as indicated. The ECLplus Western Blot Detection System (Beyotime, Shanghai, China) was employed to visualize protein bands. The antibody against ZBTB1 at 1:1000 dilution was purchased from Invitrogen (Carlsbad, CA, USA, Cat: PA-62203). The FLAG antibody at 1:5000 dilution was purchased from Sigma-Aldrich (Burlington, MA, USA, Cat: A8592). The antibodies against LDHA (Cat 19987-1-AP,1:2000), HK2 (Cat22029-1-AP, 1:2000), c-Myc (Cat 67447-1-Ig and 10828-1-AP, 1:2000), HA (Cat 51064-2-AP, 1:5000) and β-Actin (Cat 81115-1-RR, 1:2000) were purchased from Proteintech (Wuhan, China).

### 2.5. Chromatin Immunoprecipitation (ChIP)

ChIP assay was conducted using BeyoChIP™ ChIP Assay Kit (Beyotime, Shanghai, China). Briefly, 5 × 10^6^ cells were fixed with 1% formaldehyde for 10 min. After washing twice with cold PBS, cells were harvested and lysed using lysis buffer for 20 min on ice. Chromatin was sonicated to generate DNA fragments of approximately 200–800 bp. For each immunoprecipitation reaction, chromatin complexes were incubated with 2–5 μg of specific antibody or corresponding IgG control antibody overnight at 4 °C. Ten percent of the total chromatin was reserved as input control. After immunoprecipitation, reverse cross-linking and DNA purification were conducted. Quantitative PCR was used to detect target HK2 and LDHA promoter regions. A genomic negative control region was included in qPCR analysis. The enrichment of binding was calculated using the percent-input method: Percent input = 100 × 2^(Ct(input) − Ct(IP))^. Primers for the promoters of LDHA and HK2 are listed as follows: LDHA forward primer: 5′-GATCAGCCTGACCAACATGGTGA-3′, LDHA reverse primer: 5′-TGAAACGGAGTCGCTCTGTCGCC-3′, HK2 forward primer: 5′-AGGAAGAGCAAAGACCCTTGGGTG-3′, HK2 reverse primer: 5′-GCCACTCACCCTCACAGCCAGTC-3′.

### 2.6. Cell Viability Assay

Cell counting kit-8 (CCK-8) was purchased from Beyotime (Shanghai, China) and used to measure cell viability in vitro. A total of 5 × 10^3^ cells were plated into a 96-well plate. Then, 72 h later, CCK-8 was introduced to the plate and then incubated for 2 h. The microplate reader (Biolab, Atlanta, GA, USA) was used to detect the absorbance of 450 nm. To avoid potential interference of glycolytic status on CCK-8 assay readings, we also performed direct viable-cell counting as a complementary approach. In total, 1× 10^4^ cells were seeded in 12-well plates. At the indicated time points, cells were trypsinized and resuspended, and viable cell numbers were directly counted using a hemocytometer.

### 2.7. Soft Agar Assay

A 0.6% agar in complete medium was plated into a 6-well plate as a bottom layer agar. After concreting, 5 × 10^3^ cells in complete medium were mixed with 0.6% low melting agarose (agarose content is 0.3%) and were plated on the bottom layer of 0.6% agar. Then 2 mL medium was added to the top of the cell layer. Then, 2mL DMEM was added to protect the top layer from drying. Only colonies larger than 50 μm in diameter were scored. For each well, at least five random fields were imaged and quantified. The assay was performed with three independent biological replicates. Colony counting was performed in a blinded manner.

### 2.8. Co-Immunoprecipitation (Co-IP)

Co-IP assays were conducted to investigate the interaction between ZBTB1 and c-Myc in TNBC cells as previously described [38]. A total of 5 × 10^6^ cells were lysed using 0.5 mL IP buffer (50 mM Tris, 150 mM NaCl, 1 mM DTT, 0.25% NP-40 and protease inhibitor) and the samples were incubated with 3 μg anti-ZBTB1 antibody overnight at 4 °C. Then 50 μL Protein A/G agarose was used to capture antibody-containing complexes. The Protein A/G agarose was washed three times using cold-IP buffer. The beads were washed four times with cold lysis buffer to remove non-specifically bound proteins. After extensive washing, bound proteins were eluted in SDS loading buffer and subjected to Western blot analysis. To avoid interference from immunoglobulin heavy and light chains, blots were probed with a secondary antibody that exhibits minimal cross-reactivity with denatured rabbit/mouse IgG fragments; alternatively, non-reducing SDS-PAGE can be used to minimize immunoglobulin heavy/light chain interference during Western blot detection. Corresponding quantitative analysis is shown in Figure S12.

### 2.9. Mass Spectrometry Analysis

To screen potential ZBTB1-binding proteins, co-immunoprecipitation coupled with mass spectrometry (LC-MS/MS) was performed. Briefly, BT-549 cells were transfected with FLAG-ZBTB1 overexpression plasmid or empty vector control. Cell lysates were harvested and incubated with anti-FLAG magnetic beads for protein immunoprecipitation. The enriched protein complexes were collected and sent to Huijun Biotechnology (Guangzhou, China) for LC-MS/MS identification. Mass spectrometry analysis was performed using a Thermo Orbitrap mass spectrometer (Waltham, MA, USA). Raw spectral data were searched against the human UniProt database using the Mascot search engine. Stringent protein screening criteria were applied to obtain reliable interacting candidates: proteins with pep_expect < 0.05 and matched with at least two unique specific peptides. False discovery rate (FDR) correction was used to control false-positive results. Proteins specifically enriched in the FLAG-ZBTB1 group but not in the empty vector group were considered potential ZBTB1-interacting candidates.

### 2.10. RNA Immunoprecipitation (RIP) Assay

Imprint^®^ RNA Immunoprecipitation Kit (Millipore, Burlington, MA, USA) was used to identify lncRNAs that bind to ZBTB1. The assays were conducted as described previously [39]. Briefly, 1× 10^7^ cells were harvested for each RIP reaction. Samples were incubated with 3 μg anti-ZBTB1 antibody or isotype-matched IgG control overnight at 4 °C. IgG enrichment was included to subtract non-specific background binding. After immunoprecipitation, coprecipitated RNAs were extracted. RNA integrity was verified by agarose gel electrophoresis and spectrophotometric detection. Co-immunoprecipitated RNAs were extracted by a miRNeasy Mini Kit (Qiagen, Hilden, Germany) and then subjected to real-time PCR to detect the enrichment of target RNAs bound to ZBTB1. Beta-Actin transcript was used as the negative RNA control. Enriched target RNA levels were quantified by qPCR. Relative enrichment was normalized to input RNA and calculated using the percent-input method: Percent input = 100 × 2^(Ct(input) − Ct(RIP))^. Co-immunoprecipitated RNAs were extracted by a miRNeasy Mini Kit (Qiagen, Hilden, Germany) and then subjected to real-time PCR to detect the enrichment of target RNAs bound to ZBTB1.

### 2.11. Measurements of Lactate Production and Glucose Uptake

Lactate Assay Kit II and glucose uptake assay kit (Biovision, Milpitas, CA, USA) were used to detect lactate production and glucose uptake of TNBC cells. The assays were conducted as described previously [37]. Cells were seeded in a 12-well plate in complete medium. After 12 h, cells were maintained in FBS free medium for 6 h. Then, the medium was harvested and incubated with 0.5 mL reaction buffer for half an hour. The absorbance at 450 nm indicates the lactate concentration. To measure glucose uptake, glucose-starved cells were incubated with Krebs-Ringer-Phosphate-HEPES (KRPH) solution for 1 h and subsequently incubated with 2-deoxyglucose for 30 min. The following reactions were performed as the instructions. The absorbance of final reaction solution was detected at 412 nm and the absorbance indicated relative glucose uptake. Lactate production and glucose uptake were normalized to total protein content.

### 2.12. Immunohistochemistry Staining

To examine the expression of ZBTB1 in TNBC, immunohistochemistry (IHC) staining was performed using TNBC and matched normal tissues. The specimens were incubated with ZBTB1 antibody overnight at 4 °C and then reacted with anti-rabbit secondary antibody for 2 h at 37 °C. The IHC results were scored by the staining intensity and proportion of positively staining cells as described previously [37]. The IHC antibody used in this study has been previously validated for formalin-fixed, paraffin-embedded (FFPE) human breast cancer tissue in a published study [37]. All IHC staining evaluations were performed in a blinded manner without access to patient clinical information or grouping results. Immunohistochemistry (IHC) was performed with positive and negative controls included in each staining batch. The target protein was predominantly localized in the cell nucleus. The adjacent paracancerous tissues used in this study were histologically confirmed to be non-malignant by pathologists. The IHC staining score was determined by combining the staining intensity and the positive cell proportion. A pre-specified cut-off value was applied to stratify patients into high- and low-expression groups. To ensure scoring reproducibility, two independent pathologists performed the histological evaluation blindly. Interobserver agreement was calculated, and substantial consistency was achieved between the two observers.

### 2.13. Animal Experiments

This protocol was approved by the Ethical Committee of the Yichun University of Medical School Under approval number 2022 (011). Animal Care and Use Protocol complied with the ARRIVE guidelines and the U.K. Animal Act, 1986 and associated guidelines, and the EU Directive 2010/63/EU for animal experiments. Female 4–6-week-old BALB/c nude mice (BALB/c-nu) were obtained from Hunan SJA Laboratory Animal Co., Ltd. Animals were maintained under SPF housing conditions. BALB/c-nu mice harbor a spontaneous *Foxn1* mutation leading to T-cell deficiency, with intact innate immunity. No genetic manipulation was applied to these animals. All mice were clinically examined after arrival; only healthy mice were used for experiments. Research staff received special training in animal care and handling. Each cage had a maximum of four mice, and cage positions were randomly arranged and regularly rotated to avoid location bias. All mice were housed under specific pathogen-free (SPF) conditions in a climate and light-controlled facility at 23 ± 1 °C with a 12 h light/dark cycle. Mice were monitored daily for health issues such as weight loss, loss of mobility, etc. Health issues and tumor overgrowth (the long diameter > 12mm) were set as the humane endpoint. Once the endpoint was reached, mice were immediately euthanized by gradually increasing the concentration of CO_2_ in the anesthesia chamber. All efforts were made to minimize animal discomfort. The sample size for subcutaneous xenograft experiments was chosen empirically according to prior in-house experience and the published literature for similar tumor-bearing mouse models. Tumor volume was calculated using the following formula: Volume = (length × width^2^)/2.

A total of 2 × 10^6^ cells in Matrigel (Corning, Corning, NY, USA) were injected into the second pad fat of female BALB/c nude mice (4–6 weeks old). Mice were assigned into groups and subjected to the treatments as indicated. When tumors reached 80 mm^3^, mice were intraperitoneally administered vehicle or doxorubicin at a dose of 2.5 mg/kg body weight every other day for 2 weeks. Breast tumor growth was examined twice a week. Upon reaching the endpoint, mice were sacrificed and excised tumors were weighed. In this study, no mouse died before the humane endpoint. No obvious clinical toxicity or body weight loss was observed during treatment. Tumor-volume measurements were carried out in randomized animal order. Animal cages housing mice of distinct experimental groups were periodically rotated on the housing rack to mitigate positional differences in light, temperature and ventilation. A double-blind design was employed in all experiments. Principal investigator Jiajun Cui was aware of the group allocation at the different stages of mouse experiments. Other investigators were blinded to mouse group assignments during all stages of experimentation, animal handling, and data analysis. ZBTB1 or SAP30L-AS1 expression in the excised tumor tissues was confirmed (Figure S11).

### 2.14. Human Tissue Analysis

A total of 110 formalin-fixed paraffin-embedded cancer and para-cancerous tissues of TNBC were obtained from the 1st Affiliate Hospital of Yichun University and Jiangxi Cancer Hospital. This study was approved by the institutional review boards of Yichun University and Jiangxi Cancer Hospital. All patients enrolled in the study signed written informed consent. All cases were female with 32–79 years of age (mean age: 53.2 years) and the median follow-up was 75 months (range, 4–76 months). ZBTB1 protein expression in these tissue samples was detected using IHC. All patients included in this study were diagnosed with triple-negative breast cancer (TNBC) based on standard immunohistochemical criteria (ER-negative, PR-negative, HER2-negative). Clear inclusion and exclusion criteria were defined for patient enrollment. Patients with incomplete clinical data, preoperative anti-tumor therapy, or concurrent malignancies were excluded.

### 2.15. Statistical Analysis

Statistical analyses and plotting were performed using GraphPad Prism 7.0. All tests were two-sided, and *p* < 0.05 was considered statistically significant. Unless noted, experiments were repeated three times, and data are presented as mean ± SD. Normality was assessed by D’Agostino–Pearson test and variance homogeneity by Levene’s test. Two-group comparisons were performed using unpaired Student’s *t*-test or Mann–Whitney *U* test. One-way ANOVA with Tukey’s post hoc test (Bonferroni multiplicity adjustment) or Kruskal–Wallis with Dunn’s post hoc test was used for multiple-group comparisons. For survival analysis, Kaplan–Meier curves and the log-rank test were used; univariate Cox regression calculated HR with 95% CI, and a number-at-risk table was shown at 0, 20, 40, 60, and 80 months. IC_50_ values were fitted by four-parameter logistic non-linear regression.

## 3. Results

### 3.1. ZBTB1 Is Downregulated in TNBC

**Figure 1:**
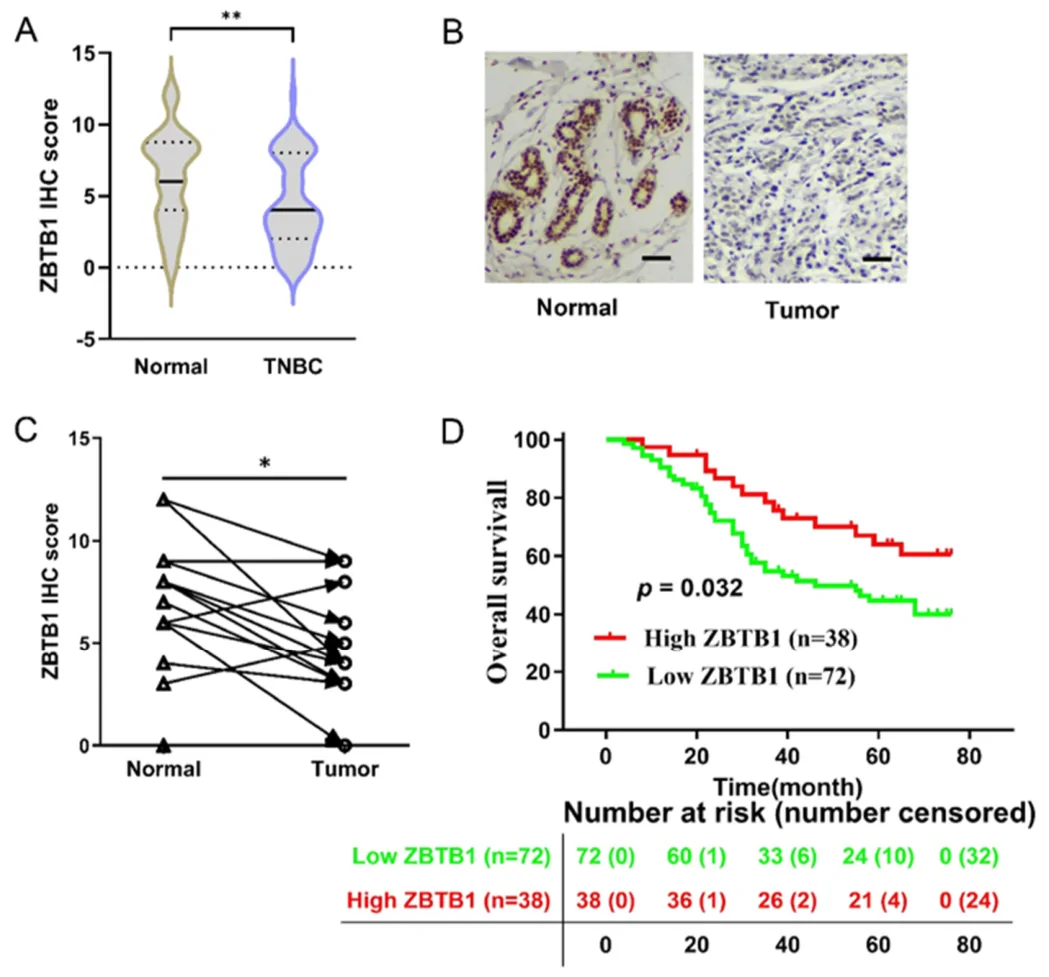
ZBTB1 expression is reduced in TNBC. (**A**,**B**) IHC staining was used to detect ZBTB1 expression in TNBC and normal tissues (*n* = 60). The scale bar is 50 μm. *p* = 0.002, Cohen’s *d* = 0.57. (**C**) ZBTB1 expression in 15 pairs of breast cancer and adjacent normal tissues was detected using IHC assay (*p* = 0.022, Cohen’s *d* = 0.88). These 15 tumor samples are included in the 60 TNBC tumor specimens described above. (**D**) The association between overall survival and ZBTB1 expression was detected in 110 TNBC patients, which included the 60 samples described above by Kaplan–Meier method. *: *p* < 0.05, **: *p* < 0.01.

Our previous study shows that ZBTB1 is downregulated at mRNA level in TNBC [37]. To confirm the downregulation of ZBTB1 at protein level in TNBC, we investigated the alteration of ZBTB1 protein expression in TNBC. Immunohistochemistry (IHC) assays indicated that ZBTB1 expression is reduced at protein level in TNBC compared with normal breast tissues (Figure 1A–C). More importantly, the downregulation of ZBTB1 indicates a shorter overall survival in TNBC patients (hazard ratio:1.909; 95% CI:1.108-3.29) (Figure 1D).

### 3.2. ZBTB1 Suppresses TNBC Cell Proliferation and Tumor Growth

**Figure 2:**
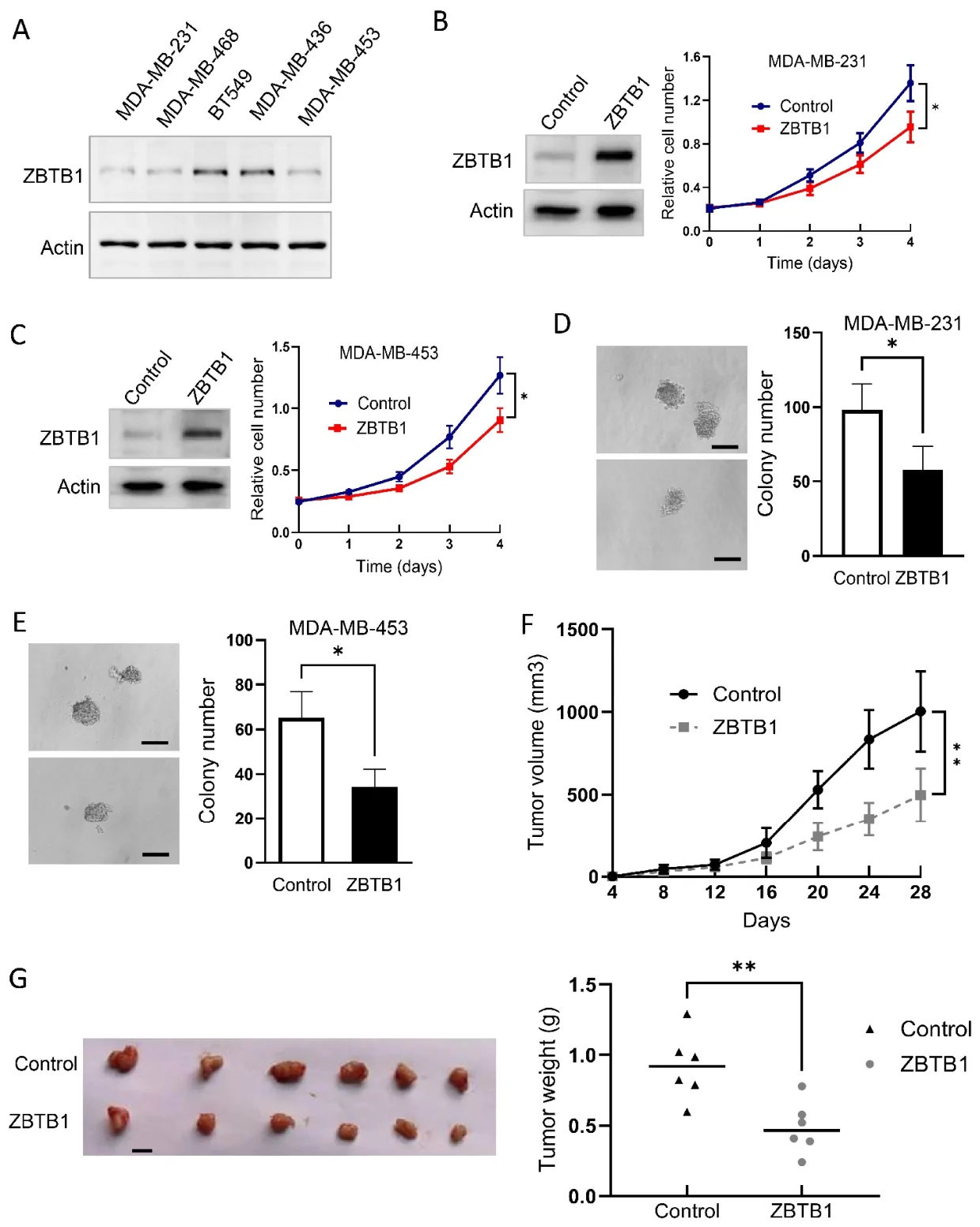
ZBTB1 reduces cell proliferation and tumor growth. (**A**) ZBTB1 expression in TNBC cells was measured using Western blot. (**B**,**C**) CCK-8 assay in MDA-MB-231 and MDA-MB-453 cells overexpressing ZBTB1. Exact *p* = 0.032 (**B**) and Exact *p* = 0.013 (**C**). (**D**,**E**) Soft agar assay for cell growth in ZBTB1 overexpressing MDA-MB-231 and MDA-MB-453 cells. The scale bar is 0.1 mm. Exact *p* = 0.042 (**D**) and Exact *p* = 0.025 (**E**). (**F**) The effect of ZBTB1 on the growth of MDA-MB-231-derived tumors in nude mice. Nude mice were injected with MDA-MB-231 cells that express ZBTB1 or control (*n* = 6 per group, two groups). The tumor volumes are shown as mean ± SD. *p* = 0.0016, Cohen’s *d* = 2.47. (**G**) Tumor weight was measured at the end of experiments. The scale bar is 10 mm. *p* = 0.0053, Cohen’s *d* = 2.05. *: *p* < 0.05, **: *p* < 0.01.

Next, we studied the impact of ZBTB1 on TNBC cell proliferation. First, we investigated ZBTB1 expression in different TNBC cell lines. We found that ZBTB1 exhibited significantly lower levels in MDA-MB-231 and MDA-MB-453 compared with BT-549 and MDA-MB-436 cells (Figure 2A). Then, we infected these two cell lines with ZBTB1-expressing lentivirus. CCK-8 assays showed that ZBTB1 overexpression significantly suppressed MDA-MB-231 and MDA-MB-453 cell proliferation (Figure 2B,C and Figure S1A,B), whereas ZBTB1 silencing significantly promoted BT-549 and MDA-MB-436 cell proliferation (Figure S1C). We also found that ZBTB1 reduced anchorage-independent cell growth (Figure 2D,E).

To further study the effect of ZBTB1 on tumor growth, we injected ZBTB1-expressing MDA-MB-231 cells into nude mice. As a result, overexpression of ZBTB1 reduced tumor growth in nude mice (Figure 2F,G). Collectively, these findings suggest that ZBTB1 negatively regulates TNBC cell proliferation and tumor growth.

### 3.3. ZBTB1 Suppresses Glycolysis in TNBC Cells

**Figure 3:**
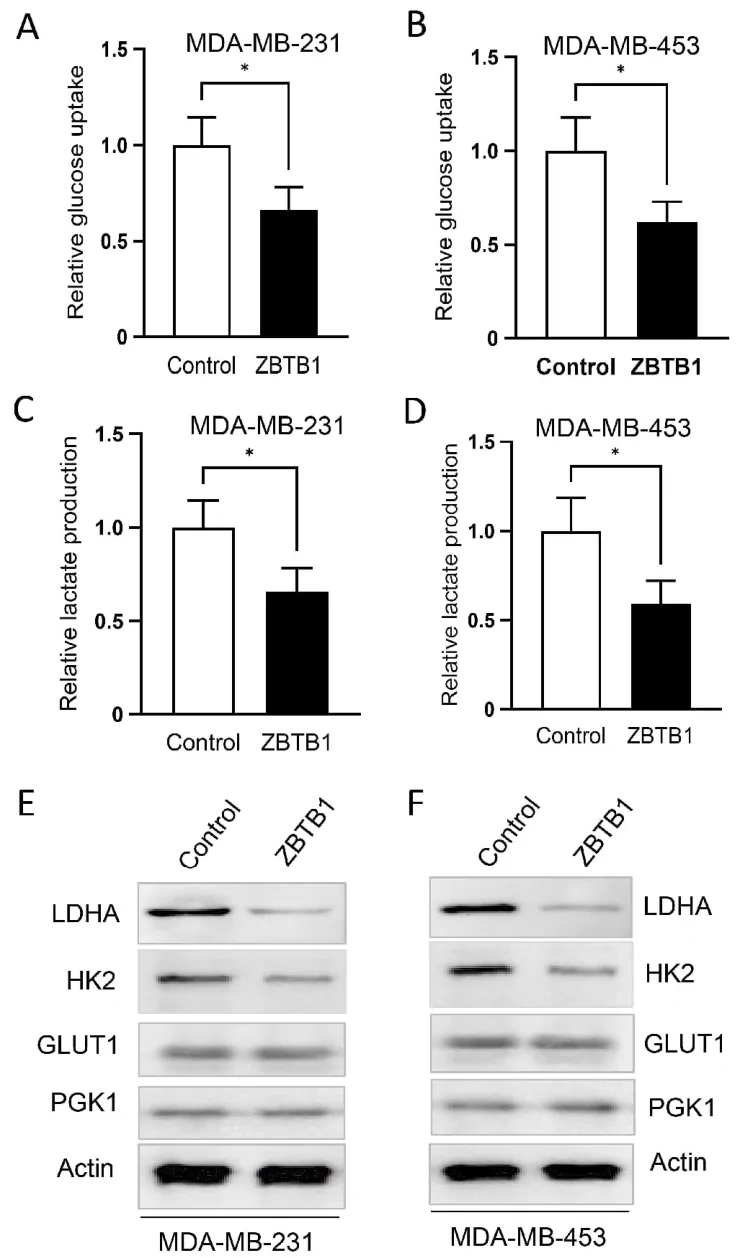
ZBTB1 suppresses glycolysis in TNBC cells. (**A**,**B**) Glucose uptake was detected in MDA-MB-231 and MDA-MB-453 cells which overexpressed ZBTB1. Exact *p* = 0.037 (**A**) and Exact *p* = 0.044 (**B**). (**C**,**D**) Lactate release was detected in MDA-MB-231 and MDA-MB-453 cells which overexpressed ZBTB1. Exact *p* = 0.032 (**C**) and Exact *p* = 0.042 (**D**). (**E**,**F**) The effect of ZBTB1 on HK2 and LDHA expressions was examined using Western blot in MDA-MB-231 and MDA-MB-453 cells which expressed ZBTB1 or control plasmids. *: *p* < 0.05.

Our previous study has shown that ZBTB1 regulates glycolysis in ER positive breast cancer [37]. To study the impact of ZBTB1 on glycolysis in TNBC cells, we measured lactate production and glucose uptake in MDA-MB-231 and MDA-MB-453 cells. As a result, ZBTB1 overexpression significantly reduced lactate production and glucose uptake in MDA-MB-231 and MDA-MB-453 cells (Figure 3A–D). We also found that ZBTB1 overexpression reduced the protein levels of HK2 and LDHA, two key enzymes of glycolysis (Figure 3E,F). Moreover, knockdown of ZBTB1 increased lactate production and glucose uptake in BT-549 and MDA-MB-436 cells (Figure S2A–D). These data suggested that ZBTB1 suppresses glycolysis in TNBC cells.

### 3.4. ZBTB1 Regulates Chemotherapy Resistance in TNBC Cells

**Figure 4:**
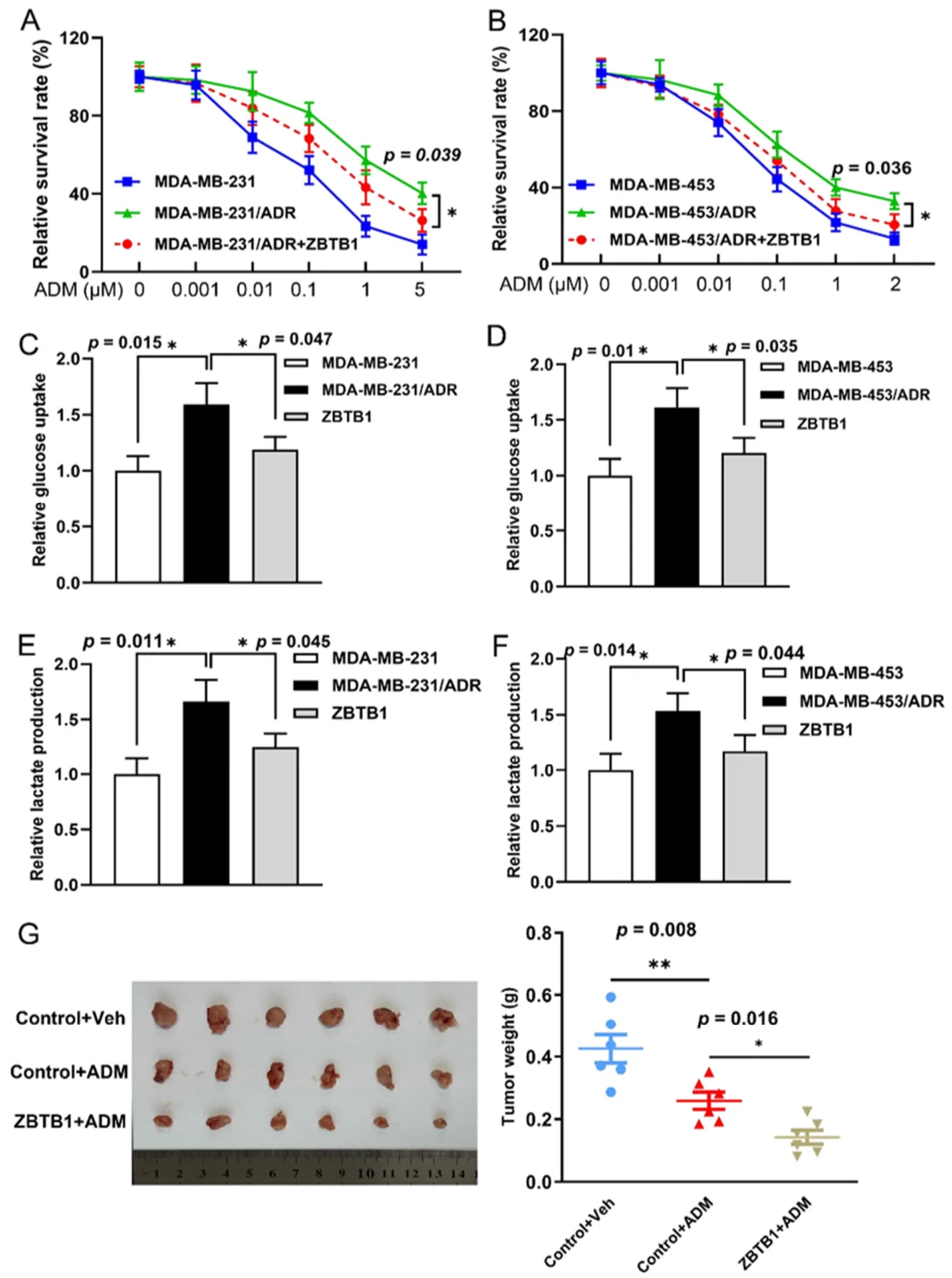
ZBTB1 regulates chemotherapy resistance in TNBC cells. (**A**,**B**) MDA-MB-231/ADR and MDA-MB-453/ADR cells were infected by lentivirus expressing ZBTB1 and then treated with Adriamycin for 72 h. Cells were collected and subjected to CCK-8 assays. Each group’s viability is normalized to its own untreated control (set as 100%). (**C**,**D**) Glucose uptake was measured in cells described as above. (**E**,**F**) Lactation production was measured in cells described as above. (**G**) The effect of ZBTB1 on the tumor growth exposure to Adriamycin. Nude mice were injected with ZBTB1-overexpressing MDA-MB-231/ADR or control cells. Three weeks later, mice were treated with Adriamycin or vehicle as indicated (n = 6 per group, three groups). Tumor weights were measured at the endpoint of experiments. *: *p* < 0.05, **: *p* < 0.01.

The association between glycolysis and chemotherapy resistance in TNBC has been well established [23,25]. Thus, we carried out a series of experiments to study the role of ZBTB1 in chemotherapy resistance in TNBC. First, the Adriamycin-resistant MDA-MB-231/ADR and MDA-MB-453/ADR cells were developed. Compared with the corresponding parental cells, the Adriamycin IC_50_ increased from 0.104 μM to 1.807 μM (95% CI for the difference: 1.24–2.16) in MDA-MB-231/ADR cells, with a resistance index (RI) of 17.4. For MDA-MB-453/ADR cells, the Adriamycin IC_50_ rose from 0.063 μM to 0.371 μM (95% CI for the difference: 0.22–0.39), yielding an RI of 5.1. We then investigated if ZBTB1 might be involved in Adriamycin resistance in these resistant cells. We observed that ZBTB1 overexpression sensitized MDA-MB-231/ADR and MDA-MB-453/ADR cells to Adriamycin. Compared with control cells, ZBTB1 overexpression decreased the Adriamycin IC_50_ from 1.807 μM to 0.623 μM (95% CI for the difference: −1.57–−0.79) in MDA-MB-231/ADR cells. For MDA-MB-453/ADR cells, the Adriamycin IC_50_ decreased from 0.371 μM to 0.132 μM (95% CI for the difference: −0.32–−0.24) (Figure 4A,B). Direct cell counting confirmed these results (Figure S3A,B). Then, we measured lactate production and glucose uptake in MDA-MB-231/ADR and MDA-MB-453/ADR cells. As a result, the increases in glucose uptake and lactate release were observed in resistant cells. Importantly, we found that ZBTB1 overexpression could diminish the increases in lactate production and glucose uptake in resistant cells (Figure 4C–F). To investigate the impact of ZBTB1 on Adriamycin sensitivity in vivo, nude mice were injected with ZBTB1-expressing MDA-MB-231/ADR or control cells and then treated with Adriamycin or vehicle. ZBTB1 overexpression further strengthened the inhibitory effect of Adriamycin on tumor growth, indicating that ZBTB1 enhances the sensitivity of breast tumors to Adriamycin therapy (Cohen’s *d* = 1.89) (Figure 4G and Figure S3C,D). These data suggested that ZBTB1 sensitizes TNBC cells to chemotherapy in vitro and in vivo.

### 3.5. ZBTB1 Interacts with c-Myc and Represses Its Transcriptional Activity

**Figure 5:**
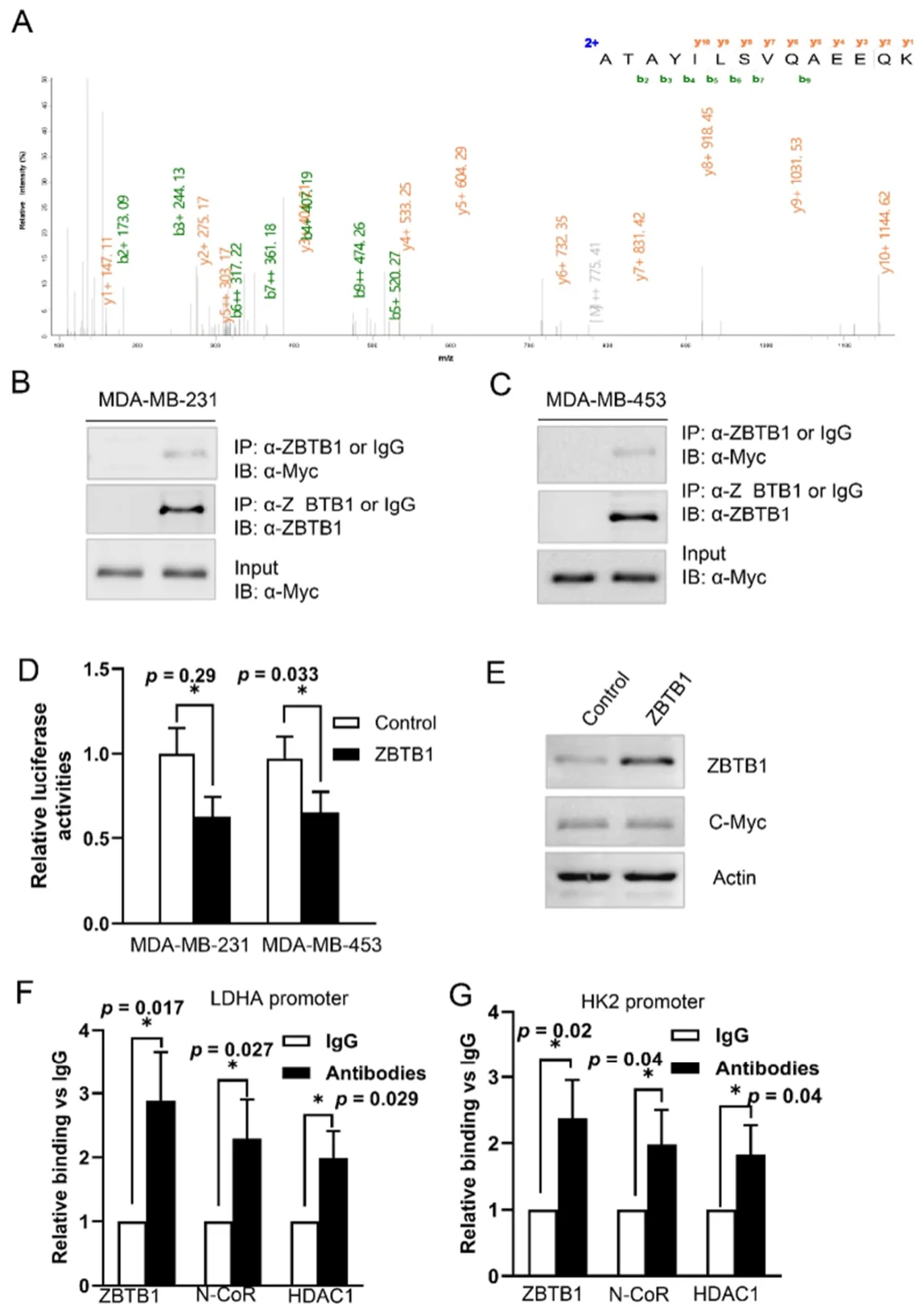
ZBTB1 interacts with c-Myc. (**A**) LC-MS/MS analysis to identify ZBTB1-binding proteins in MDA-MB-231 cells expressing FLAG-ZBTB1. The maximum peak intensity of the mass spectrum is 1 × 10^5^. (**B**,**C**) Endogenous Co-IP was carried out using anti-ZBTB1 and anti-c-Myc antibody in MDA-MB-231 and MDA-MB-453 cells. The first lane represents the IgG negative control, the second lane shows immunoprecipitation with anti-ZBTB1 antibody, and corresponding c-Myc inputs are shown at the bottom. (**D**) The effect of ZBTB1 on c-Myc transcriptional activity. MDA-MB-231 and MDA-MB-453 cells were co-transfected with ZBTB1 and c-Myc reporter gene. After 24 h, cells were collected to perform luciferase activity measurement. (**E**) The effect of ZBTB1 on c-Myc expression in TNBC cells. MDA-MB-231 cells were treated by lentivirus containing ZBTB1 and then Western blot was conducted to measure c-Myc expression. (**F**,**G**) ZBTB1 and the co-repressors recruitment on the promoters of *LDHA* and *HK2* genes in MDA-MB-231 cells were detected by ChIP assay. *: *p* < 0.05.

To gain a better understanding of the mechanism of how ZBTB1 regulates glycolysis, we performed immunoprecipitation (IP) assays in MDA-MB-231 cells which were infected with FLAG-ZBTB1 containing lentivirus or control vector and followed by LC-MS/MS analysis to identify ZBTB1-binding proteins. Using this filtering strategy, we identified 204 candidate proteins that were present in the ZBTB1-overexpressing group and completely absent in the control group. Detailed information for these proteins is listed in Supplemental Tables S1 and S2. Among these proteins, c-Myc attracted our interest due to its key roles in TNBC (Figure 5A). Next, we detected the possible interaction between ZBTB1 and c-Myc. The endogenous c-Myc protein in MDA-MB-231 and MDA-MB-453 cells was immuno-precipitated using anti-ZBTB1 antibody and the following Western blot showed that c-Myc interacted with ZBTB1 (Figure 5B,C). For reverse co-immunoprecipitation (reverse Co-IP), anti-c-Myc antibody was used to immunoprecipitate endogenous protein complexes, followed by immunoblotting with anti-ZBTB1 antibody. The results confirmed the interaction between c-Myc and ZBTB1 (Figure S4A,B).

Then, we studied the role of ZBTB1 in c-Myc transcriptional activity. As a result, we found that ZBTB1 overexpression suppressed c-Myc transcriptional activity in MDA-MB-231 and MDA-MB-453 cells (Figure 5D). In addition, we investigated the effect of ZBTB1 on c-Myc expression and found that ZBTB1 overexpression produced no effect on c-Myc expression in TNBC cells (Figure 5E).

To study the molecular mechanisms under the regulation of c-Myc transcriptional activity by ZBTB1, we performed chromatin immunoprecipitation (ChIP) assays to detect the ZBTB1 and its associated co-repressors including N-CoR (nuclear receptor co-repressor 1) and HDAC1 (histone deacetylase 1) recruitment to the c-Myc-binding region of *LDHA* and *HK2* promoters in MDA-MB-231 and MDA-MB-453 cells. As a result, we found that ZBTB1 and its co-repressors could be recruited to the promoters of *LDHA* and *HK2* in MDA-MB-231 and MDA-MB-453 cells (Figure 5F,G and Figure S5A,B). Real-time PCR assays showed that ZBTB1 suppresses the expressions of *LDHA* and *HK2* in MDA-MB-231 and MDA-MB-453 cells (Figure S5C,D). In addition, we found that ZBTB1 does not affect the recruitment of c-Myc on the promoters of *LDHA* and *HK2* in MDA-MB-231 cells (Figure S5E,F). Finally, ChIP-re-ChIP assays suggested that ZBTB1 and MYC co-occupy the promoters of *LDHA* and *HK2* in MDA-MB-231 cells (Figure S5G,H). These data indicated that ZBTB1 may suppress c-Myc transcriptional activity through interaction with c-Myc.

### 3.6. SAP30L-AS1 Facilitates the Physical Interaction Between ZBTB1 and c-Myc in TNBC Cells

**Figure 6:**
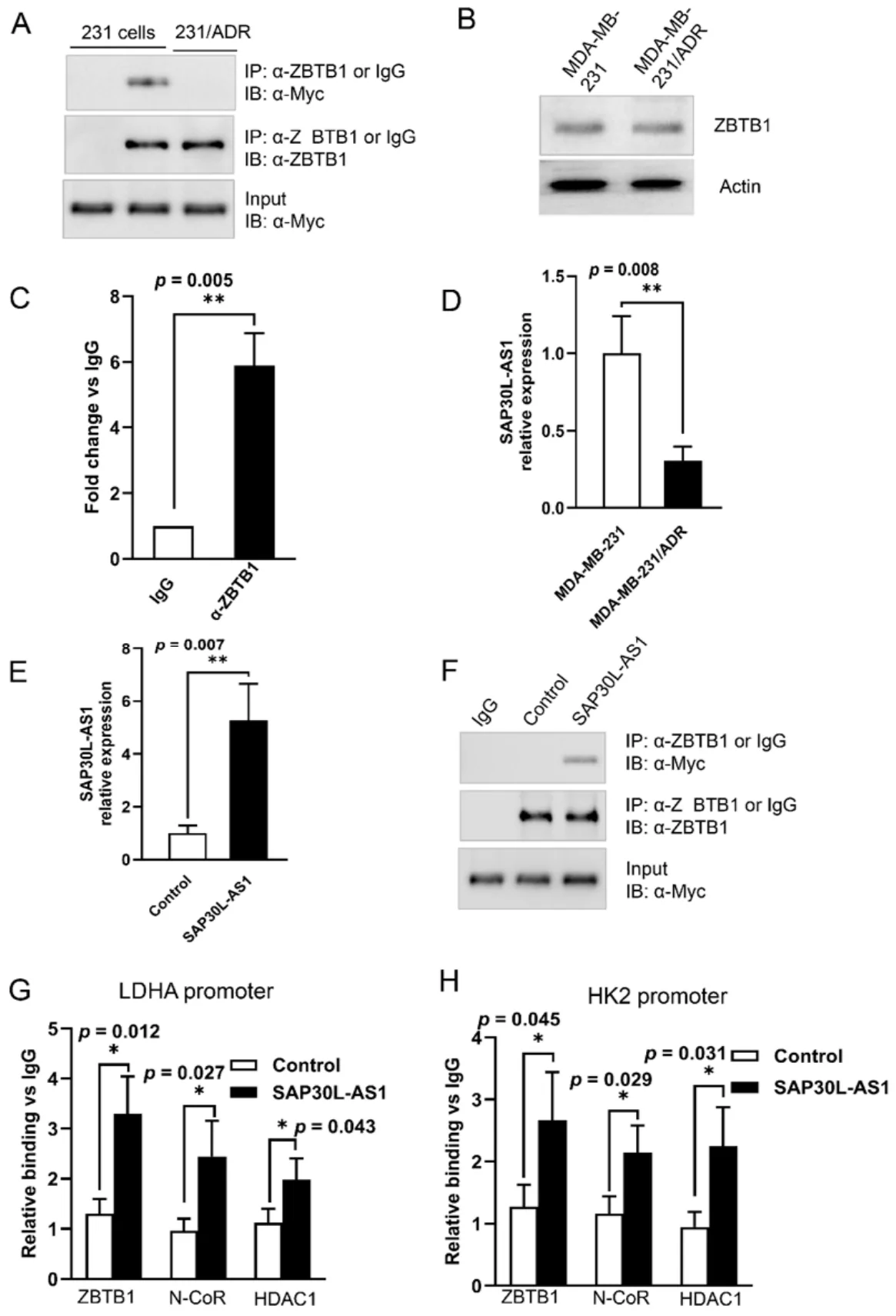
SAP30L-AS1 is required for the interaction between ZBTB1 and c-Myc in TNBC cells. (**A**) Endogenous Co-IP was carried out using ZBTB1 and c-Myc antibody in MDA-MB-231 and MDA-MB-231/ADR cells. The first lane represents the IgG negative control, the second and third lane shows immunoprecipitation with anti-ZBTB1 antibody in control and SAP30L-AS1-overexpressing cells, and the corresponding c-Myc inputs are shown at the bottom. (**B**) ZBTB1 expression was detected by Western blot in MDA-MB-231 and MDA-MB-231/ADR cells. (**C**) RIP assay was performed to identify the interaction between ZBTB1 and SAP30L-AS1 in MDA-MB-231 cells. (**D**) Relative SAP30L-AS1 expression was detected by real-time PCR in MDA-MB-231 and MDA-MB-231/ADR cells. (**E**) Relative SAP30L-AS1 expression was detected by real-time PCR in MDA-MB-231/ADR cells which were treated by lentivirus containing SAP30L-AS1 or control. (**F**) Endogenous Co-IP was carried out using ZBTB1 and c-Myc antibodies in MDA-MB-231/ADR cells which were treated by lentivirus containing SAP30L-AS1 or control plasmids. (**G**,**H**) ZBTB1 and the recruitment of its co-repressors on the promoters of *LDHA* and *HK2* genes in MDA-MB-231/ADR cells as indicated (**E**) were detected by ChIP assay. *: *p* < 0.05, **: *p* < 0.01.

To understand if the interaction with c-Myc mediates the effect of ZBTB1 on chemotherapy resistance, we performed Co-IP in Adriamycin-resistant cells and their parent cells. Remarkably, the interaction between ZBTB1 and c-Myc was readily detected in MDA-MB-231 and MDA-MB-453 but not MDA-MB-231/ADR and MDA-MB-453/ADR cells (Figure 6A and Figure S6A). Then, we detected the expression of ZBTB1 in both cell lines and observed no difference between Adriamycin-resistant cells and their parent cells (Figure 6B and Figure S6B). These results indicated that the disrupted ZBTB1–c-Myc interaction in MDA-MB-231/ADR cells does not arise from reduced ZBTB1 abundance, implying that additional regulatory molecules modulate their physical interaction.

Zinc finger motifs are well-established RNA-binding domains that bridge lncRNA–protein interactions [40]. For instance, the lncRNA LNBC3 binds the ZBTB family member BCL6 to modulate BCL6 activity in lung carcinoma [41]. Therefore, we speculated that there may exist one LncRNA that interacts with ZBTB1 and regulates its function in TNBC cells. To address this possibility, MDA-MB-231 cell lysates were treated with RNase A and we found the interaction between ZBTB1 and c-Myc was abolished in MDA-MB-231 cells. These results indicate that the interaction between ZBTB1 and c-Myc is RNA-dependent in MDA-MB-231 cells (Figure S7). catRAPID omics v2.1 was applied to predict ZBTB1-interacting LncRNAs. Then, we selected the top 50 lncRNAs ranked by score to further analyze their expression changes in breast cancer based on the TCGA database. We obtained 20 LncRNAs with altered expressions in breast cancer and then performed RNA immunoprecipitation (RIP) assay to identify ZBTB1-binding LncRNAs in BT-549 cells. As a result, we found that SAP30L-AS1 displayed the strongest binding affinity (Figure S8). The binding of ZBTB1 with SAP30L-AS1 was confirmed in MDA-MB-231 and MDA-MB-453 cells using RIP assay (Figure 6C and Figure S9A).

Therefore, we decided to carry out a series of experiments to investigate the role of SAP30L-AS1 in ZBTB1-mediated chemotherapy resistance. First, we investigated the expression of SAP30L-AS1 in MDA-MB-231/ADR and MDA-MB-453/ADR cells and we found that SAP30L-AS1 exhibited a much lower level in Adriamycin-resistant cells than their parent cells (Figure 6D and Figure S9B). Then, we infected MDA-MB-231/ADR using SAP30L-AS1 containing lentivirus and then performed Co-IP to investigate the interaction between ZBTB1 and c-Myc in MDA-MB-231/ADR and MDA-MB-453/ADR cells. As a result, SAP30L-AS1 re-expression restored the interaction between ZBTB1 and c-Myc in MDA-MB-231/ADR and MDA-MB-453/ADR cells (Figure 6E,F and Figure S9C,D). In addition, we silenced SAP30L-AS1 and found SAP30L-AS1 depletion abolished the interaction between ZBTB1 and c-Myc in MDA-MB-231 cells (Figure S9E,F). Moreover, we found that SAP30L-AS1 re-expression restored the recruitment of ZBTB1 and its co-repressors to the promoters of LDHA and HK2 in MDA-MB-231/ADR and MDA-MB-453/ADR cells (Figure 6G,H and Figure S9G,H). These findings demonstrated that SAP30L-AS1 is essential for maintaining the physical interaction between ZBTB1 and c-Myc in TNBC cells.

### 3.7. SAP30L-AS1 Regulates Glycolysis and Chemoresistance via ZBTB1 in TNBC Cells

**Figure 7:**
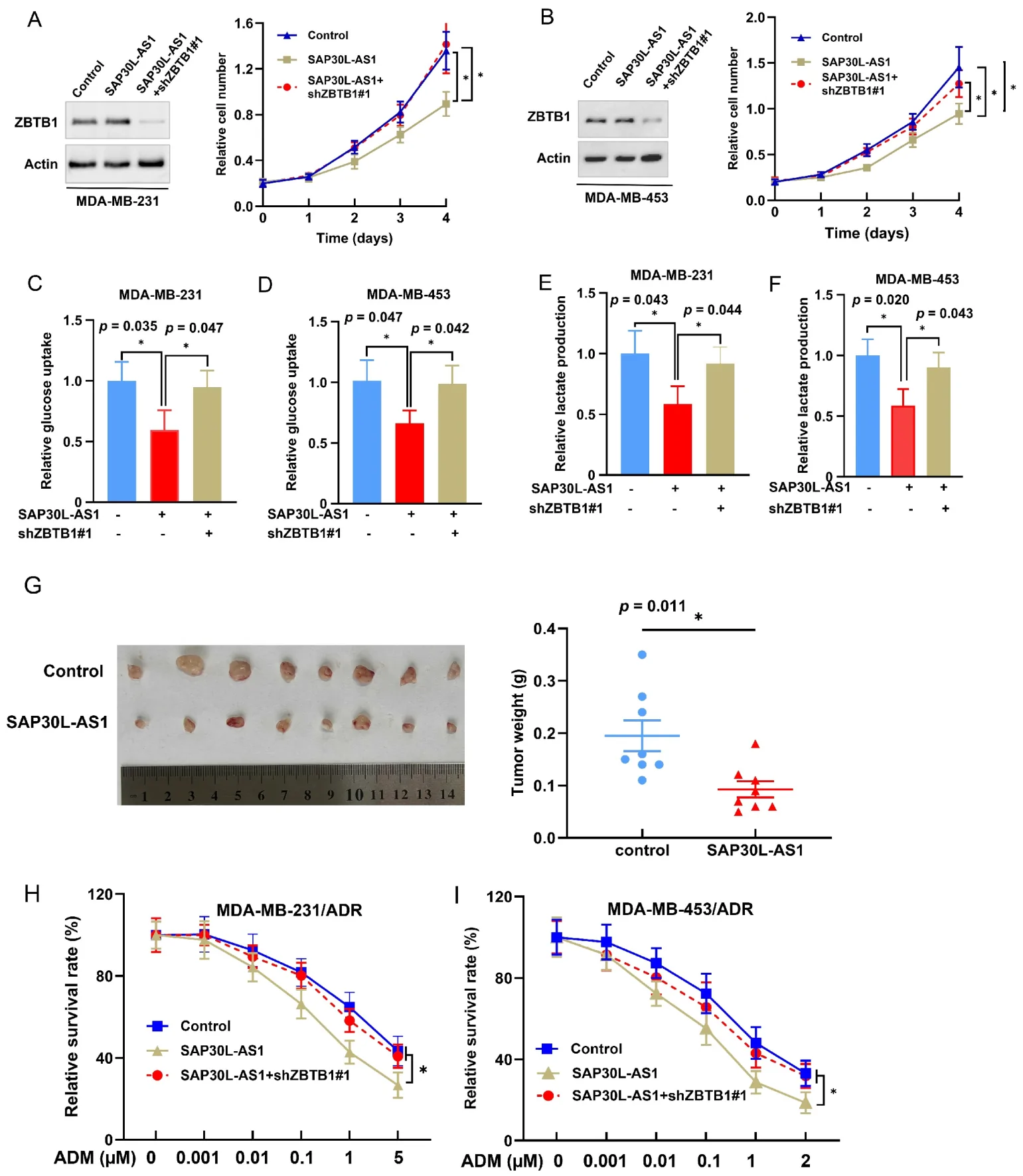
SAP30L-AS1 regulates glycolysis and chemoresistance via ZBTB1 in TNBC cells. (**A**,**B**) MDA-MB-231 and MDA-MB-453 cells were infected by lentivirus containing SAP30L-AS1 or control plasmids. Then cells were further treated by lentivirus containing ZBTB1 shRNA#1 as indicated. Western blot was used to verify ZBTB1 protein levels, and CCK-8 assays were conducted to evaluate cell proliferation. (**C**,**D**) Glucose uptake was measured in MDA-MB-231 and MDA-MB-453 cells as indicated in (**A**,**B**). (**E**,**F**) Lactate release was measured in MDA-MB-231 and MDA-MB-453 cells as indicated in panels (**A**,**B**). (**G**) The effect of SAP30L-AS1 on the growth of MDA-MB-231-derived tumors in nude mice. Nude mice were injected with MDA-MB-231 cells expressing SAP30L-AS1 or control plasmid (*n* = 8 per group, two groups). Tumor weight was measured at the end of experiments. Cohen’s *d* = 1.55. (**H**,**I**) MDA-MB-231/ADR and MDA-MB-453/ADR cells were infected by lentivirus carrying SAP30L-AS1 alone or together with shZBTB1#1 and then treated with Adriamycin for 72 h. Cells were collected and subjected to CCK-8 assay. *: *p* < 0.05.

Through Cancer Genome Atlas (TCGA) data analysis using UALCAN [42], we found that SAP30L-AS1 was significantly downregulated in TNBC compared with normal tissues (Figure S10A). To examine the role of SAP30L-AS1 in TNBC cells, we infected TNBC cells with SAP30L-AS1 containing lentivirus and then performed cell proliferation assays. CCK-8 assays revealed that SAP30L-AS1 overexpression markedly suppressed the proliferation of MDA-MB-231 (*p* = 0.015) and MDA-MB-453 cells (*p* = 0.032) and ZBTB1 knockdown largely diminished this anti-proliferative effect of SAP30L-AS1 (*p* = 0.024 in A and *p* = 0.037 in B) (Figure 7A,B). Conversely, SAP30L-AS1 knockdown obviously promoted cell proliferation in BT-549 (Figure S10B,C). To explore the role of SAP30L-AS1 in glycolysis, we detected glucose uptake and lactate production in TNBC cells. As a result, SAP30L-AS1 overexpression significantly decreased glucose uptake and lactate production in MDA-MB-231 and MDA-MB-453 cells. Moreover, ZBTB1 knockdown largely diminished the inhibitory effect of SAP30L-AS1 on glycolysis (Figure 7C–F). In addition, we found that SAP30L-AS1 silencing promoted glucose uptake and lactate production in BT-549 cells (Figure S10D,E). To study the impact of SAP30L-AS1 on tumor growth in vivo, nude mice were injected with SAP30L-AS1-expressing MDA-MB-231 cells. As a result, SAP30L-AS1 suppressed the growth of tumors derived from MDA-MB-231 cells (Figure 7G).

We then investigated if SAP30L-AS1 regulates Adriamycin resistance in TNBC cells. We found that SAP30L-AS1 overexpression sensitized MDA-MB-231/ADR (*p* = 0.038) and MDA-MB-453/ADR cells (*p* = 0.036) to Adriamycin, and ZBTB1 knockdown largely diminished this effect (*p* = 0.042 in H and *p* = 0.044 in I) (Figure 7H,I). In addition, SAP30L-AS1 depletion significantly reduced the sensitivity of MDA-MB-231 cells to Adriamycin treatment (Figure S10).

## 4. Discussion

Through this work, we have defined the key role of ZBTB1 in glycolysis and chemotherapy resistance in TNBC. First, we showed that ZBTB1 is downregulated in TNBC and ZBTB1 overexpression decreased TNBC cell proliferation and tumor growth. Second, we found that ZBTB1 modulates glycolysis and chemotherapy resistance in TNBC cells. Third, ZBTB1 interacts with c-Myc and suppresses its transcriptional activity. Finally, we found that SAP30L-AS1 binds to ZBTB1 and facilitates ZBTB1-mediated regulation of glycolysis and chemotherapy resistance in TNBC cells.

The ZBTB (zinc finger and BTB domain-containing) protein family is composed of diverse zinc finger proteins that also contain BTB (Broad-complex, Tram-track, and Bric-à-brac) domain. These proteins harbor two domains which are essential for their transcriptional regulatory functions: the N-terminal BTB domain primarily mediates protein–protein interactions and facilitates the recruitment of corepressors such as N-CoR, SMRT, Sin3a, and HDACs, while the C-terminal zinc finger domain enables sequence-specific binding to the regulatory regions of target genes [43,44,45,46]. The ZBTB proteins commonly act as transcriptional repressors through interacting with corepressors via their BTB domain. They exert diverse and critical roles in many biological processes, including cell fate decision, lineage commitment, DNA repair, hematopoietic stem cell fate determination and tumorigenesis [45,46,47]. ZBTB1 was initially identified as a critical transcriptional regulator governing lymphoid and partial myeloid development [31,32,34]. Our group’s study suggests that ZBTB1 functions as a novel tumor repressor and regulates tamoxifen resistance in ER^+^ breast cancer. ZBTB1 exhibits a much lower level in TNBC than ER^+^ breast cancer, highlighting its key role in TNBC [37]. Therefore, it is worth investigating the role of ZBTB1 in TNBC. In the present study, we found that ZBTB1 protein expression is significantly decreased and this downregulation is associated with poor outcome in TNBC. ZBTB1 overexpression suppresses cell proliferation and tumor growth. Moreover, ZBTB1 overexpression sensitizes TNBC cells to chemotherapy in vitro and in vivo. These data suggest that ZBTB1 is a tumor suppressor in TNBC. However, ZBTB1 has been shown to be upregulated in pancreatic carcinoma and the upregulation indicates worse prognosis. This phenomenon indicates that ZBTB1 acts as a tumor suppressor or promoter depending on cancer type.

Chemotherapy resistance represents the primary obstacle to TNBC treatment. An increasing number of studies suggest that metabolic reprogramming plays a critical role in chemotherapy resistance in TNBC. TNBC cells exhibit the highest level of glycolysis in all types of breast cancer [13,48]. Our previous study shows that ZBTB1 regulates tamoxifen resistance through suppressing glycolysis in ER^+^ breast cancer [37]. Here, we also found that ZBTB1 suppresses glycolysis and regulates chemotherapy resistance in TNBC cells. ZBTB1 commonly exerts its function through other transcriptional factors [37,49]. Therefore, we performed LC-MS/MS analysis to identify ZBTB1-binding proteins. Among these candidate proteins, c-Myc attracted our interest due to its key roles in TNBC [50,51]. Accumulating studies suggest that c-Myc is the key regulator of cellular metabolism such as glycolysis through promoting glycolytic gene expression. C-Myc-activated metabolic reprogramming is essential for chemotherapy resistance in TNBC [48,52]. Thus, it is worthwhile to explore the co-regulators of c-Myc involved in metabolic reprogramming in TNBC cells. Herein, our Co-IP results confirmed a physical interaction between ZBTB1 and c-Myc in TNBC cells. ZBTB1, together with its associated co-repressors N-CoR and HDAC1, can be recruited to the c-Myc binding sites on the promoters of c-Myc downstream glycolytic genes, including HK2 and LDHA. As a transcriptional repressor, ZBTB1 ultimately represses c-Myc-driven glycolysis in TNBC cells. However, further rescue experiments with c-Myc overexpression are required to formally verify that ZBTB1 regulates glycolysis and chemoresistance through c-Myc.

**Figure 8:**
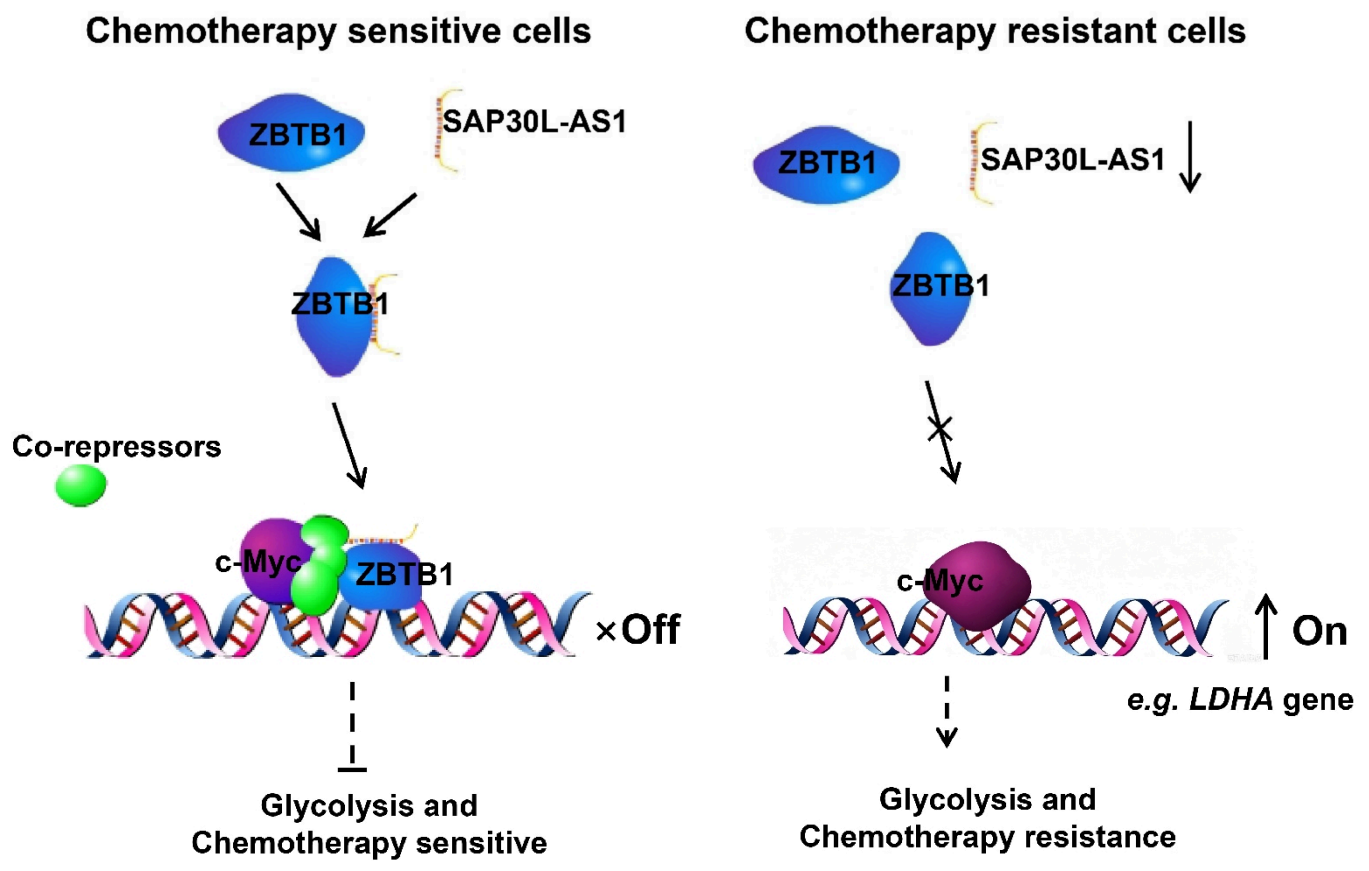
The proposed model for the actions of SAP30L-AS1/ZBTB1 on aerobic glycolysis and chemoresistance in TNBC cells. In chemotherapy-sensitive cells, SAP30L-AS1 binds ZBTB1. Subsequently, ZBTB1 is recruited to c-Myc binding sites in the promoters of glycolytic genes where it further recruits co-repressors such as N-CoR and HDAC1. Through this action, the expression of glycolytic genes is reduced and thereby aerobic glycolysis is followed suppressed. In chemotherapy-resistant cells, the loss of SAP30L-AS1 expression prevents ZBTB1 from occupying c-Myc binding sites; this may ultimately leads to elevated aerobic glycolysis and the acquisition of Adriamycin resistance. Solid arrows indicate validated regulatory steps. Crossed arrows represent inhibited molecular interactions. Dashed lines indicate incompletely validated regulatory steps. Upward and downward arrows mark increased and decreased gene/transcript expression, respectively.

Of note, ZBTB1 expression remains unchanged but loses the capacity to interact with c-Myc in Adriamycin-resistant cells (MDA-MB-231/ADR and MDA-MB-453/ADR cells). This phenomenon implies the involvement of additional regulatory mediators. Zinc finger motifs are well-characterized RNA-binding domains that mediate lncRNA–protein interactions [40]. For example, the lncRNA LNBC3 physically associates with a ZBTB protein BCL6 and promotes the oncogenic function of BCL6 in lung carcinoma [41]. Our data suggest that RnaseA treatment abolishes the interaction of ZBTB1 and c-Myc. Inspired by these findings, we hypothesized that an uncharacterized lncRNA may interact with ZBTB1 to govern its biological activity in triple-negative breast cancer (TNBC) cells. Through bioinformatic analysis and RIP verification, a lncRNA SAP30L-AS1 was identified to bind with ZBTB1 and is required for the interaction between ZBTB1 and c-Myc in TNBC cells. SAP30L-AS1 expression is dramatically reduced in MDA-MB-231/ADR and MDA-MB-453/ADR cells compared to their parent cells. Consequently, ZBTB1 is not able to bind c-Myc and recruit corepressors to the promoters of glycolytic genes in MDA-MB-231/ADR and MDA-MB-453/ADR cells. Thus, resistant cells exhibit enhanced glycolysis and are resistant to Adriamycin treatment (Figure 8). However, further rescue experiments with c-Myc overexpression are required to confirm whether c-Myc mediates the oncogenic effects of the ZBTB1/SAP30L-AS1 axis. Moreover, patient-derived TNBC models and ZBTB1 knockout mouse models are required to confirm this pathway.

It has been shown that SAP30L-AS1 is upregulated in prostate cancer. SAP30L-AS1 binds the promoter of *SAP30L* and represses *SAP30L* transcription. By modulating SAP30L expression, SAP30L-AS1 facilitates prostate cancer cell growth [53]. However, we observed that SAP30L-AS1 is downregulated in TNBC and suppresses TNBC cell growth in vitro and in vivo. These findings indicate that SAP30L-AS1 exerts either oncogenic or tumor-suppressive effects in a cancer-type-dependent manner. In this study, we found that SAP30L-AS1 regulates chemotherapy resistance and glycolysis via interaction with ZBTB1. Our RIP assays support their interaction in vitro. However, their direct binding still needs to be verified by RNA pull-down assays. We attempted RNA pull-down assays for SAP30L-AS1 to detect its binding with ZBTB1. The full-length SAP30L-AS1 transcript is relatively long, which hampers the performance of RNA pull-down experiments. Truncated fragment-based RNA pull-down assays will therefore be conducted in future work to validate the direct interaction between SAP30L-AS1 and ZBTB1.

Given the essential role of ZBTB1 in governing the development of T, B, and NK cells, we speculated that the SAP30L-AS1/ZBTB1 axis may act as a critical bridge connecting malignant tumor cells with the tumor immune microenvironment [31,34,36]. Of note, this hypothesis remains speculative and cannot be tested using nude mouse models. Future work employing ZBTB1-knockout mouse models will be required to investigate whether this axis mediates the interplay among chemoresistance, aerobic glycolysis, and immune escape in TNBC.

## 5. Conclusions

Collectively, our data suggest that ZBTB1 regulates glycolysis and chemotherapy resistance through interacting with c-Myc in TNBC cells. The lncRNA SAP30L-AS1 is required for maintaining the ZBTB1–c-Myc complex, and this regulatory circuit may be involved in chemotherapy resistance. Our findings suggest that the SAP30L-AS1/ZBTB1 axis may represent a potential therapeutic target for overcoming chemotherapy resistance in TNBC, although the underlying molecular details remain to be fully elucidated.

## Data Availability

Data will be made available on request.

## Supplementary Materials

The following supporting information can be downloaded at https://www.mdpi.com/article/10.3390/cancers18193156/s1. Figure S1: The of ZBTB1 on TNBC cell growth; Figure S2: ZBTB1 silencing promotes glycolysis in TNBC cells; Figure S3: ZBTB1 regulates chemotherapy resistance in TNBC cells; Figure S4: Reverse Co-IP in MDA-MB-231 cells; Figure S5: The recruitment of ZBTB1 and co-repressors on the promoters of *LDHA* and *HK2* genes in MDA-MB-453 cells; Figure S6: ZBTB1 is not able to bind c-Myc in MDA-MB-453/ADR cells; Figure S7: The interaction between ZBTB1 and c-Myc is RNA-dependent in MDA-MB-231 cells.; Figure S8: Screening of ZBTB1-interacting lncRNAs in BT549 cells; Figure S9: SAP30L-AS1 is required for the interaction between ZBTB1 and c-Myc in TNBC cells; Figure S10: TSAP30L-AS1 silencing promotes cell growth and glycolysis in TNBC cells; Figure S11: ZBTB1 and SAP30L-AS1 expression detection in excised tumor in this study; Figure S12: Densitometric quantification of co-IP immunoblots was performed using ImageJ software (version 2.9.0/1.53t); Figure S13: The plasmid maps used in this study; Table S1: ZBTB1 interacting proteins by MS; Table S2: Flag NC MS; Supporting information: STR authentication and mycoplasma testing reports for cell lines.

## Abbreviations

The following abbreviations are used in this manuscript:

TNBC	Triple Negative Breast Cancer
ER	Estrogen Receptor
LDHA	Lactate Dehydrogenase A
ZBTB	Zinc Finger and Broad-Complex, Tramtrack, and Bric-à-Brac
HK2	Hexokinase 2
ZBTB1	Zinc Finger and Broad-Complex, Tramtrack, and Bric-à-Brac 1
CCK-8	Cell Counting kit-8
ChIP	Chromatin Immunoprecipitation
Co-IP	Co-immunoprecipitation
RIP	RNA Immunoprecipitation
IHC	Immunohistochemistry
N-CoR	Nuclear Receptor Co-repressor 1
HDAC1	Histone Deacetylase 1
