# Supplementary material for: ZBTB1 Regulates Glycolysis and Chemotherapy Resistance Through Interacting with c-Myc in Triple Negative Breast Cancer
Source: Cancers (Basel). 2026 Sep 29;18(19):3156. doi: 10.3390/cancers18193156 (PMC13664490; doi:10.3390/cancers18193156)
Supplement: Supplementary file 1 [file cancers-18-03156-s001.zip › Supplementary data.pdf]

# Supplementary materials

**Materials and Methods:**

*SAP30L-AS1 knockdown in MDA-MB-231 and BT549 cells*

LncRNA smart silencer was obtained from Ribobio (Guangzhou, China) for SAP30L-AS1. The lncRNA smart silencer consists of a pool of three small interference RNAs (siRNAs) and three anti-sense oligonucleotides (ASO), which can effectively knock down both nuclear and cytoplasmic lncRNAs. The siRNA sequences are as follows: CACACTTACTTGGAGTATT, GCCATTTGTTCTGCCATAA, GCTCATCACTGTCTCTCTT. The ASO sequences are as following: GGGATATAGAGGTGGACAAA, AGGAAAACCGTATCAGCACA, AGGCCAATGAGACTCAACCT. The smart silencer was transfected into BT549 cells using Lipofectamine® RNAiMAX reagent (Invitrogen, Carlsbad, CA, USA). 24 h later, cells were harvested for real-time PCR to detect SAP30L-AS1 expression. The sequences of primers used were as follows: 5′-ATCTCGGCTCACCGCAACCT-3′ and 5′-AATGACCCAAATGCACGTCCT-3′.

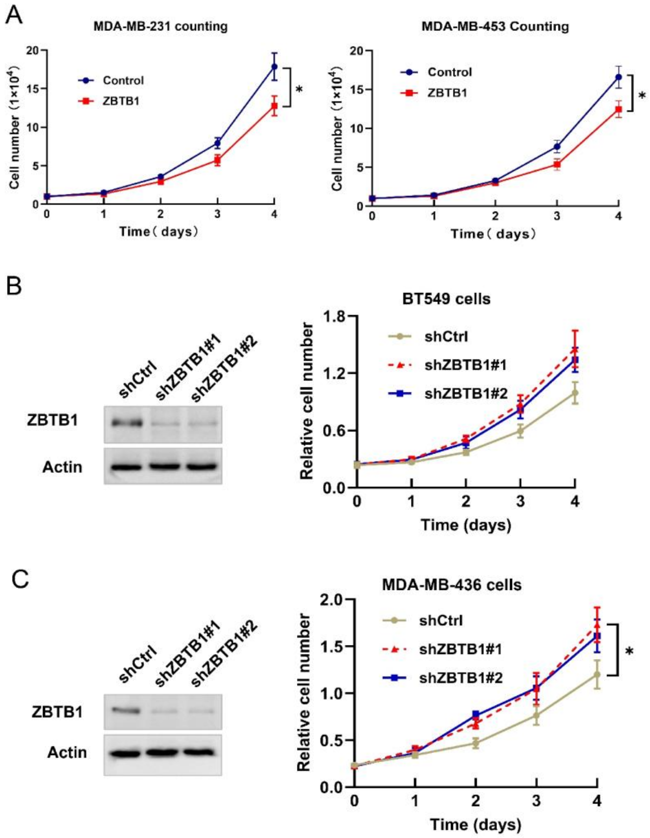


**Supplementary Figure S1. The of ZBTB1 on TNBC cell growth.** (**A**) and (**B**) MDA-MB-231 and MDA-MB-453 cells were infected with lentivirus carrying ZBTB1 or control. Then, cells were seeded in 12-well plates and cell numbers were directly counted at the indicated time. (**C**) BT549 and MDA-MB-436 cells were infected with lentivirus carrying ZBTB1 shRNA#1 and shRNA#2 and verified ZBTB1 expressions by western blot. CCK-8 assay was then performed to detect the effect of ZBTB1 silencing on cell proliferation. *: $p < 0.05$.

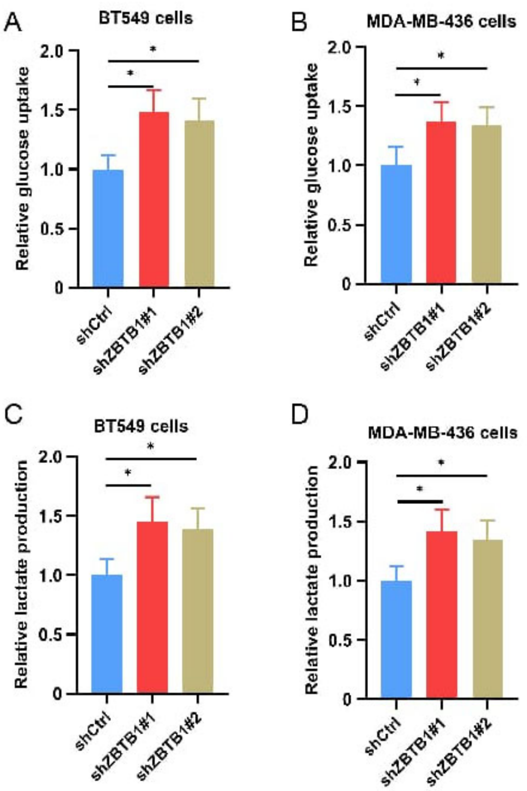


**Supplementary Figure S2. ZBTB1 silencing promotes glycolysis in TNBC cells.** (**A,B**) Glucose uptake was detected in BT549 and MDA-MB-436 cells which were infected with lentivirus carrying ZBTB1 shRNA#1 and shRNA#2. (**C,D**) Lactate production was detected in BT549 and MDA-MB-436 cells which were infected with lentivirus carrying ZBTB1 shRNA#1 or shRNA#2. *: $p < 0.05$.

A

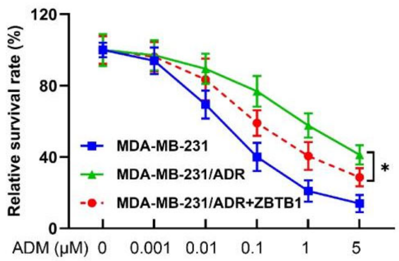


B

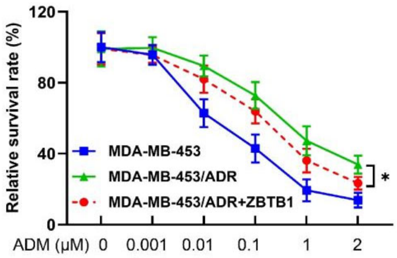


C

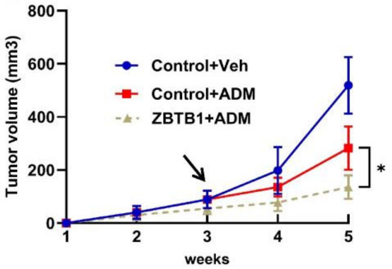


D

| Control+Veh | Control+ADM | ZBTB1+ADM |
|---|---|---|
| 0.593 | 0.354 | 0.183 |
| 0.505 | 0.312 | 0.155 |
| 0.437 | 0.226 | 0.118 |
| 0.371 | 0.283 | 0.224 |
| 0.36 | 0.194 | 0.095 |
| 0.287 | 0.186 | 0.081 |

**Supplementary Figure S3. ZBTB1 regulates chemotherapy resistance in TNBC cells.** (**A,B**) MDA-MB-231 and MDA-MB-453 cells were infected with lentivirus carrying ZBTB1 or control. Then, cells were seeded in 12-well plates and treated with Adriamycin for 72 h. Cell numbers were directly counted after being trypsinized and resuspended. (**C,D**) The effect of ZBTB1 on the tumor growth exposure to Adriamycin. Nude mice were injected with ZBTB1 overexpressing MDA-MB-231/ADR or control cells. Three weeks later, mice were treated with Adriamycin or vehicle as indicated The tumor volumes are shown at mean ± SD(C). Tumor weight was measured at the end of experiments (D). *: $p < 0.05$.

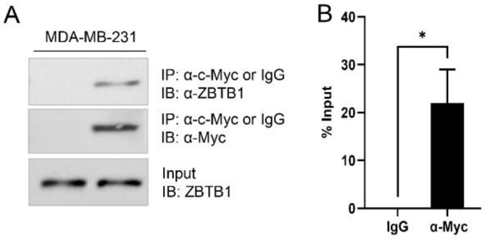


**Supplementary Figure S4: Reverse Co-IP in MDA-MB-231 cells.** (**A**) Endogenous co-immunoprecipitation (Co-IP) was performed using anti-c-Myc antibody. The precipitated protein complex was immunoblotted with anti-ZBTB1 antibody to verify the presence of ZBTB1 within the c-Myc-containing complex. (**B**) Band intensities were semi-quantitatively analyzed by grayscale scanning using ImageJ software to evaluate immunoprecipitation efficiency. The signal intensity of ZBTB1 in Co-IP products was normalized to the corresponding input sample to calculate precipitation efficiency.

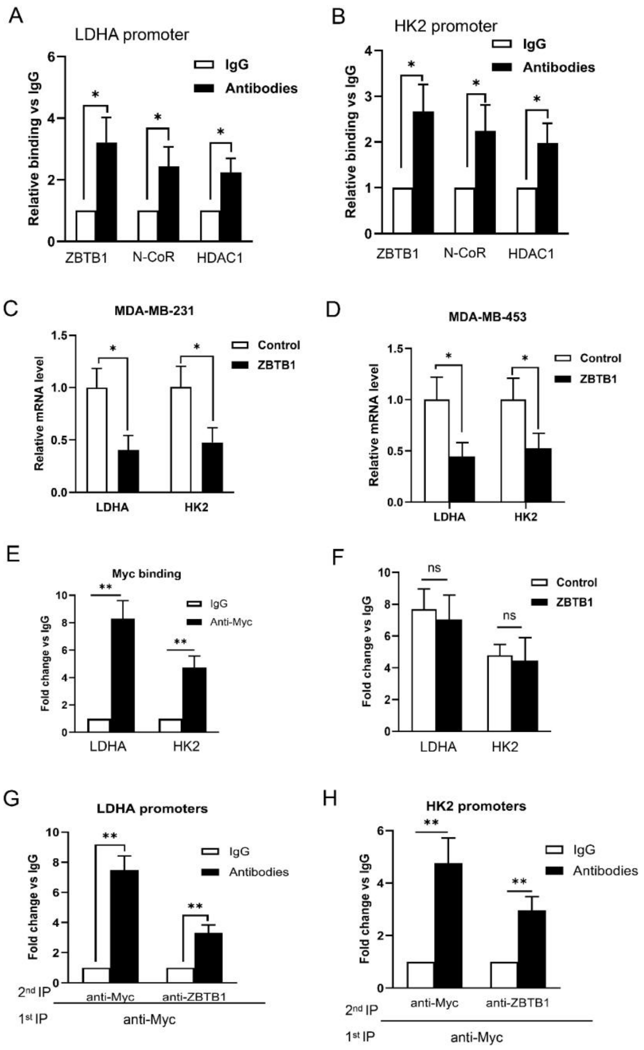


**Supplementary Figure S5. The recruitment of ZBTB1 and co-repressors on the promoters of *LDHA* and *HK2* genes in MDA-MB-453 cells.** (**A**,**B**) MDA-MB-453 cells were harvested and then ChIP assays were performed using ZBTB1, N-CoR and HDAC1 antibodies to detect the recruitment of ZBTB1 and its co-repressors on the promoters of *LDHA* and *HK2* genes. (C,**D**) MDA-MB-231 and MDA-453 cells which expressing ZBTB1 or control were harvested and then real-time PCR assays were performed to detect the expressions of *LDHA* and *HK*2. *: $p < 0.05$. (**E**) MDA-MB-231 cells were harvested and then ChIP assays were performed using c-Myc antibodies to detect the recruitment of c-Myc on the promoters of *LDHA* and *HK2* genes. (**F**) MDA-MB-231 cells which expressing ZBTB1 or control were harvested and then ChIP assays were performed using c-Myc antibodies to detect the recruitment of c-Myc on the promoters of *LDHA* and *HK2* genes. (**G**,**H**) MDA-MB-231 cells were harvested and then ChIP-re-ChIP assays were performed using c-Myc antibodies to detect the interaction of c-Myc and ZBTB1 on the promoters of *LDHA* and *HK2* genes. *: $p < 0.05$. **: $p < 0.01$.

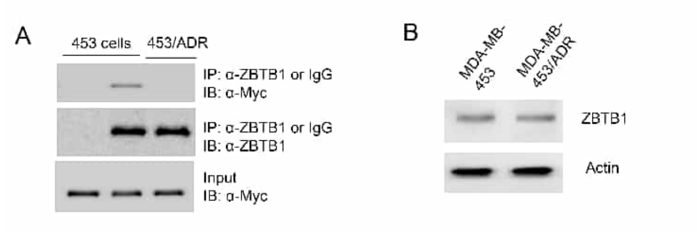


**Supplementary Figure S6: ZBTB1 is not able to bind c-Myc in MDA-MB-453/ADR cells.** (**A**) Endogenous Co-IP was carried out using ZBTB1 and c-Myc antibody in MDA-MB-453 and MDA-MB-453/ADR cells. (**B**) ZBTB1 expression was detected by Western blot in MDA-MB-453 and MDA-MB-453/ADR cells.

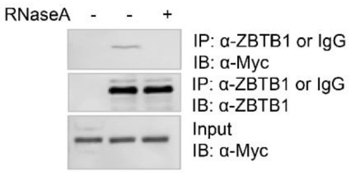


**Supplementary Figure S7: The interaction between ZBTB1 and c-Myc is RNA-dependent in MDA-MB-231 cells.** MDA-MB-231 cell lysates were treated with RNase A (20 μg/mL) or vehicle control for 30 min, followed by endogenous Co-IP using anti-ZBTB1 and anti-c-Myc antibodies.

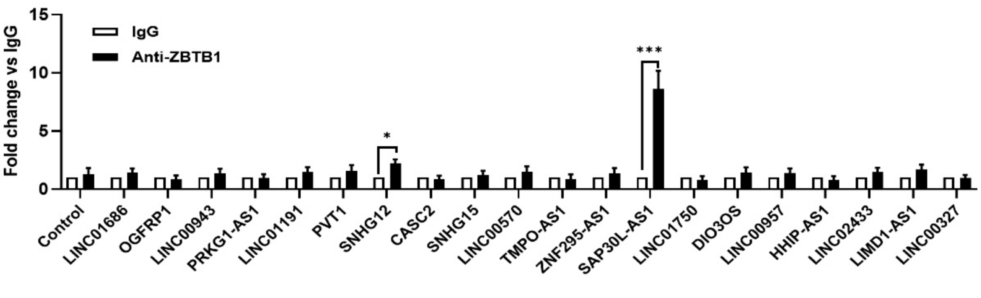


**Supplementary Figure S8. Screening of ZBTB1-interacting lncRNAs in BT549 cells.** BT549 cells were harvested and then subjected to RNA immunoprecipitation (RIP) assay to detect the interaction of candidate lncRNAs with ZBTB1. *: $p < 0.05$, ***: $p < 0.001$.

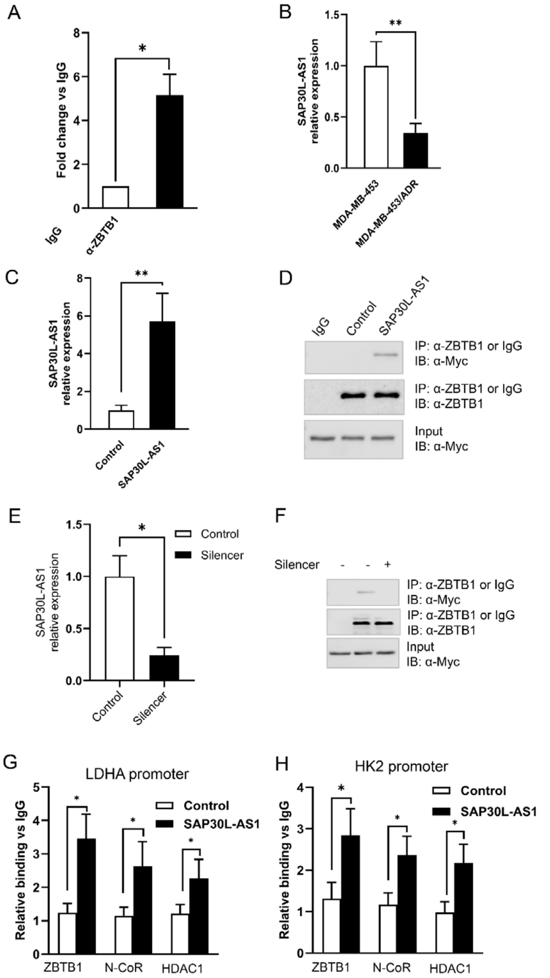


**Supplementary Figure S9. SAP30L-AS1 is required for the interaction between ZBTB1 and c-Myc in TNBC cells.** (**A**) RIP assay was performed to identify the interaction between ZBTB1 and SAP30L-AS1 in MDA-MB-453 cells. (**B**) Relative SAP30L-AS1 expression was detected by Real-time PCR in MDA-MB-453

and MDA-MB-453/ADR cells. (**C)** Relative SAP30L-AS11 expression was detected by Real-time PCR in MDA-MB-231/ADR cells which were treated by lentivirus containing SAP30L-AS1 or control. (**D**) Endogenous Co-IP was carried out using ZBTB1 and c-Myc antibodies in MDA-MB-231/ADR cells which were treated by lentivirus containing SAP30L-AS1 or control plasmids. (**E,F**) Endogenous Co-IP was carried out using ZBTB1 and c-Myc antibodies in MDA-MB-231 cells which were transfected by SAP30L-AS1 smart silencer or control. (H,**I**) ZBTB1 and the co-repressors recruitments on the promoters of *LDHA* and *HK2* genes in MDA-MB-231/ADR cells as indicated as (E) were detected by ChIP assay. *: $p < 0.05$, **: $p < 0.01$.

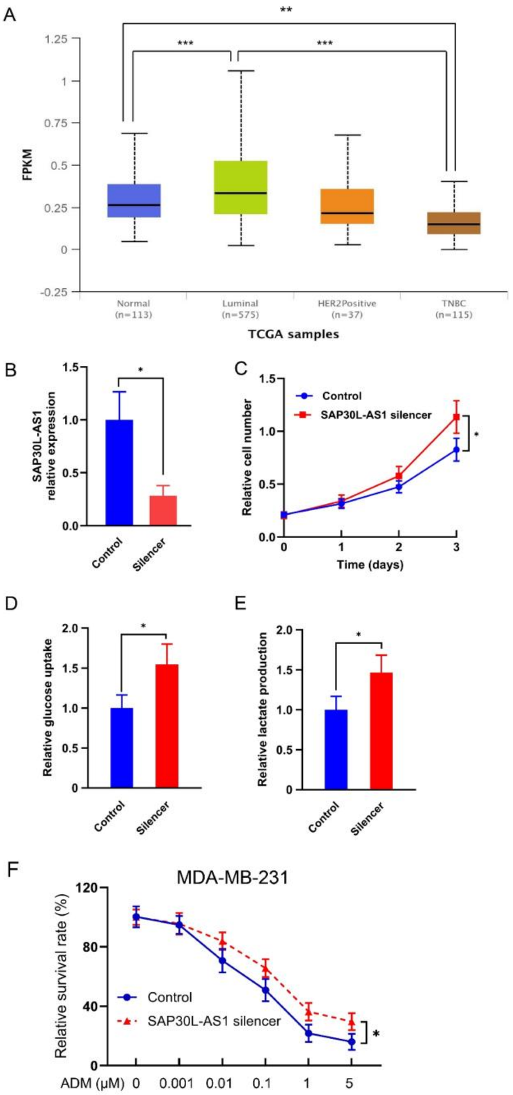


**Supplementary Figure S10. SAP30L-AS1 silencing promotes cell growth and glycolysis in TNBC cells.** (**A**) *ZBTB1* mRNA levels in normal tissues and breast tumors including major sub-types were analyzed in TAGC database. (**B**) The effect of SAP30L-AS1 smart silencer on the SAP30L-AS1 expression in BT549 cells. BT549 cells were transfected with SAP30L-AS1 smart silencer or control. 48 h later, cells were harvested for real-time PCR. (**C**) BT549 cells were transfected with SAP30L-AS1 smart silencer or negative

control. 72 h later, CCK-8 assays were performed to detect the effect of SAP30L-AS1 silencing on cell proliferation. (**D**,**E**) BT549 cells were transfected with SAP30L-AS1 smart silencer or negative control. 72 h later, glucose uptake and lactate release were detected. (**F**) The effect of SAP30L-AS1 smart silencer on the Adriamycin response in MDA-MB-231 cells. MDA-MB-231 cells were transfected with SAP30L-AS1 smart silencer or control and then treated with Adriamycin for 72h. Cells were collected and subjected to CCK-8 assay. *: $p < 0.05$, **: $p < 0.01$.

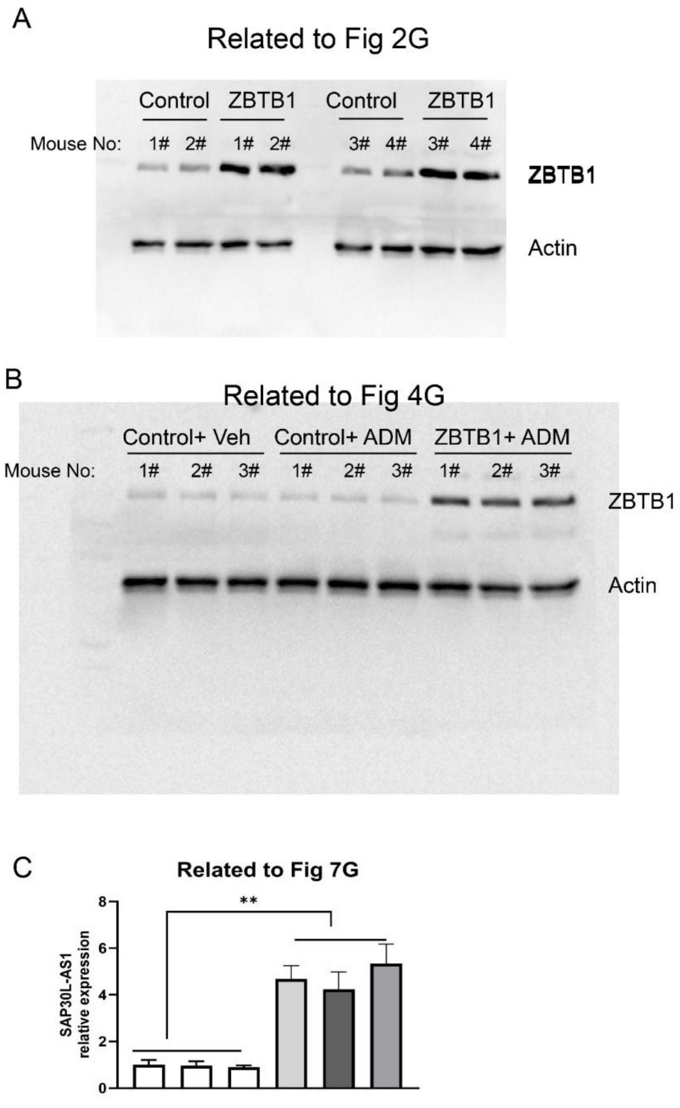


**Supplementary Figure S11. ZBTB1 and SAP30L-AS1 expression detection in excised tumor in this study.** (**A**) ZBTB1 expression using western blot in tumors from Figure 2G. (**B**) ZBTB1 expression using western blot in tumors from Figure 4G. (**C**) SAP30L-AS1 expression using real-time PCR in tumors from Figure 7G.

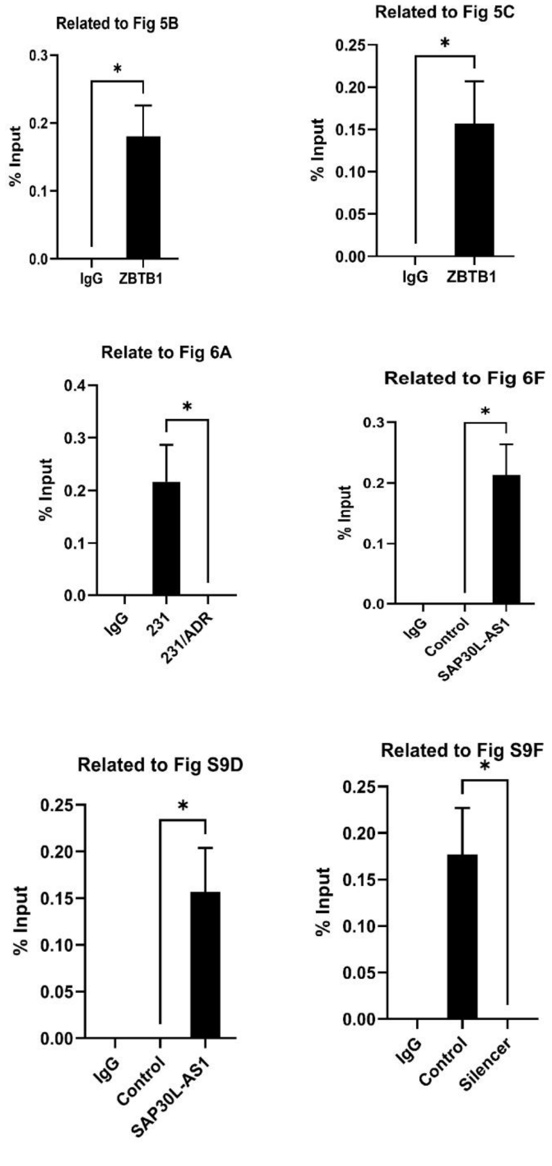


**Supplementary Figure S12. Densitometric quantification of co-IP immunoblots was performed using ImageJ software.**

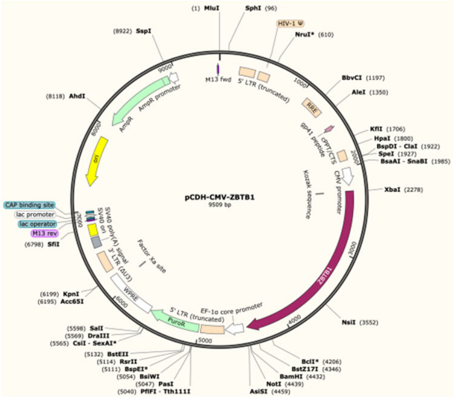


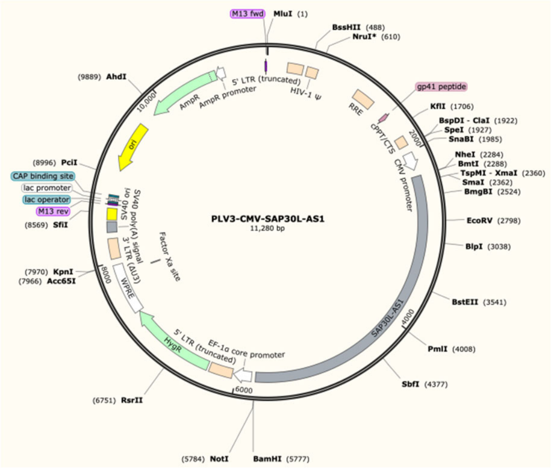


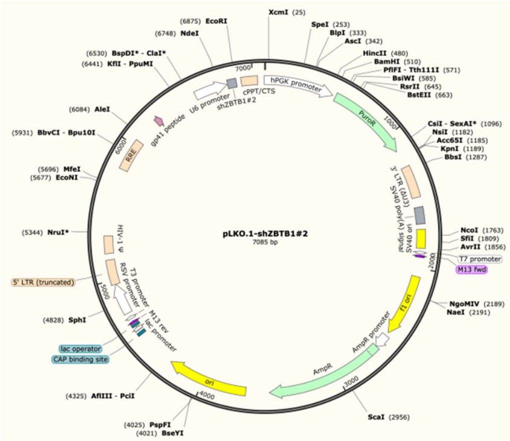


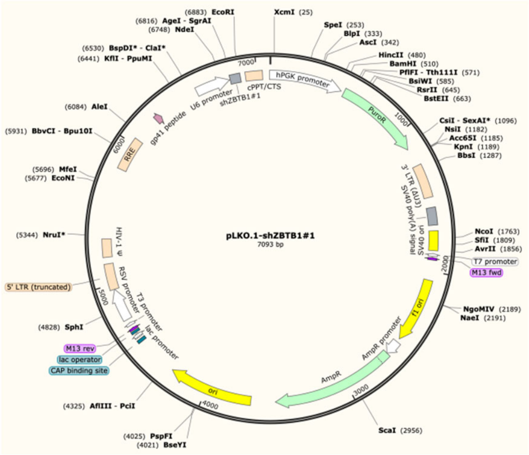


**Supplementary Figure S13. The plasmid maps used in this study.**
