# Supplementary material for: ZBTB1 Regulates Glycolysis and Chemotherapy Resistance Through Interacting with c-Myc in Triple Negative Breast Cancer
Source: Cancers (Basel). 2026 Sep 29;18(19):3156. doi: 10.3390/cancers18193156 (PMC13664490; doi:10.3390/cancers18193156)

Mycoplasma Detection Report

essential information

Report number	20240605_01
Sample Name	Cell culture supernatant
Surveillance project	Mycoplasma detection
Test method	Quantitative PCR (Dye Method)

Method Introduction

☆ Based on a quantitative real-time PCR platform, a single reaction utilizes the fluorescent dye SYBR Green.

I enables quantitative detection of Mycoplasma DNA.

☆ The experimental procedure is straightforward; the entire amplification reaction is performed under a closed-tube system, effectively preventing false-positive results caused by common product contamination in PCR experiments. Additionally, the amplification results of negative reference materials are used for quality control to identify potential false positives.

☆ This method offers higher sensitivity than the PCR method and the colorimetric method.

Quality Control

1、 Positive reference standard detection channel (FAM, blue), exhibiting a standard "S" -shaped amplification curve; CT value:
21.51/21.08 – Positive quality control established.

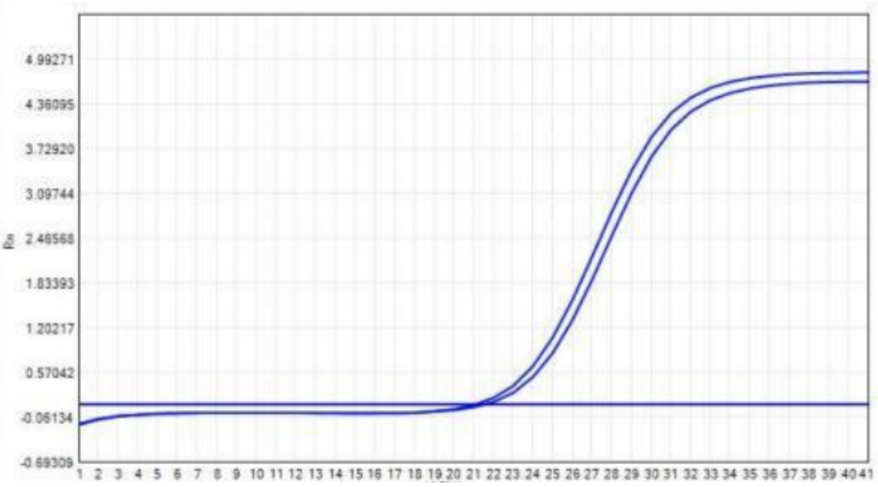

2、 The negative reference sample detection channel (FAM, blue) showed no amplification signal over 32 reaction cycles; the CT value was NoCt/NoCt, confirming the validity of the negative quality control.

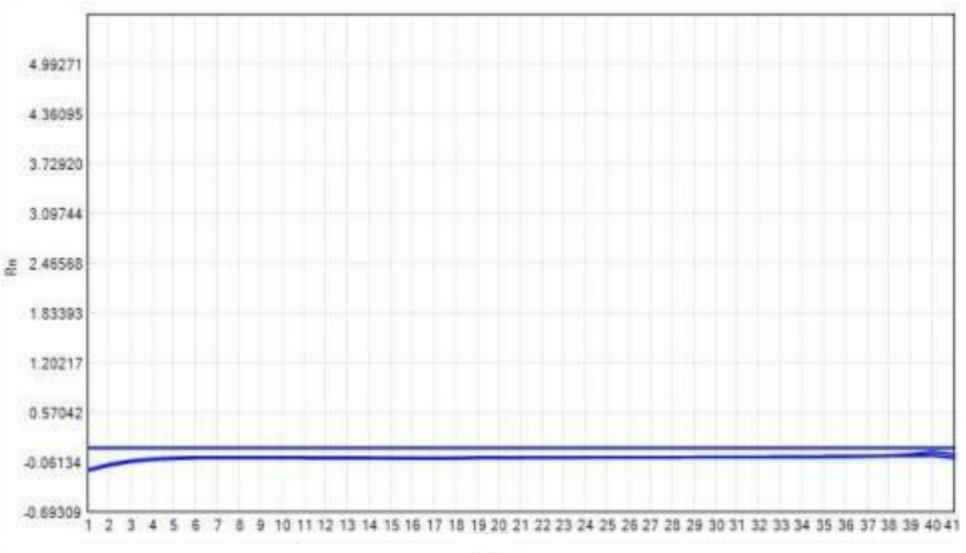

Detection Result

1、 Qualitative Standards

Detection Channel (FAM) CT Value Range	Qualitative findings
Ct≤19	Strongly positive
19<Ct≤30	Positive
30<Ct⩽32	Weakly positive – requires verification
Ct>32	Negative

Note: When Ct value equals NOCt, it indicates that no amplification signal was detected throughout the PCR reaction; this result is classified as negative.

2、 Sample test results

①The MDA-MB-231 sample detection channel (FAM, blue) showed no amplification signal over 32 reaction cycles; the CT value was NoCt/36.17, and the result was classified as negative.

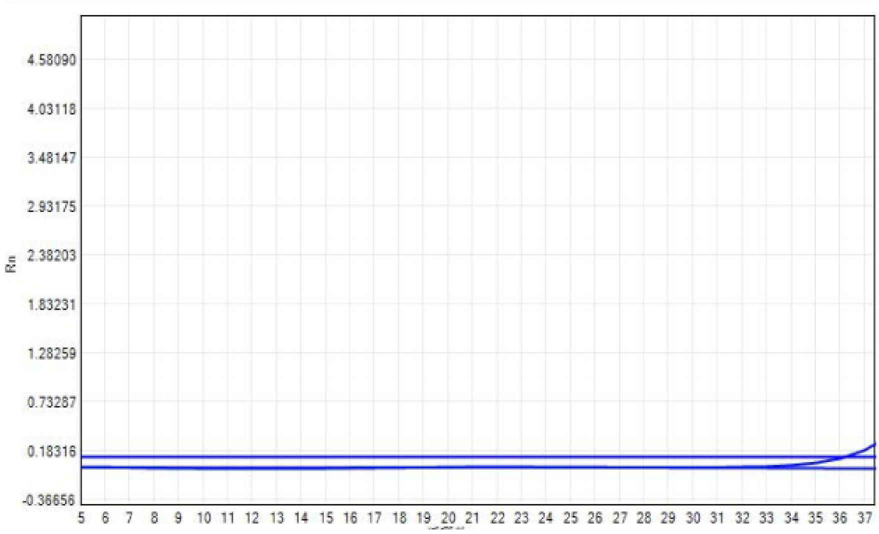

The MDA-MB-453 sample detection channel (FAM, blue) showed no amplification signal over 32 reaction cydles; the CT value was 34.41/ NoCt, and the result was classified as negative.

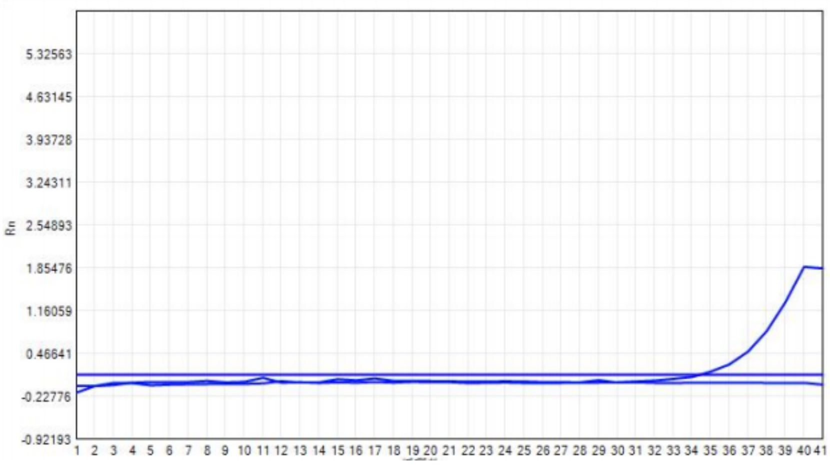

The BT-549 sample detection channel(FAM, blue) showed no amplffcation signal over 32 reation cycles; the CT value was 35.68/ 35.27- Test Result: Negative.

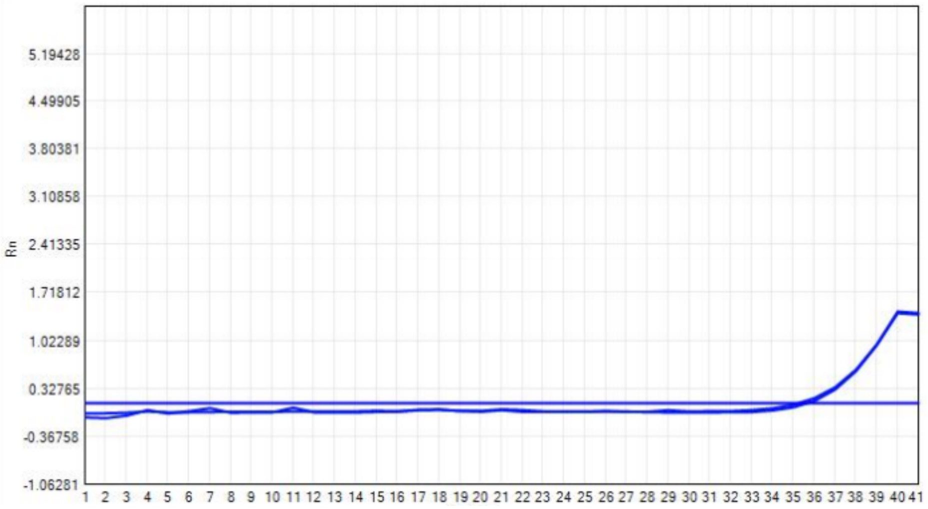

④The MDA-MB-436 sample detection channel (FAM, blue) showed no amplification signal over 32 reaction cycles; the CT value was NoCt/NoCt, and the result was classified as negative.

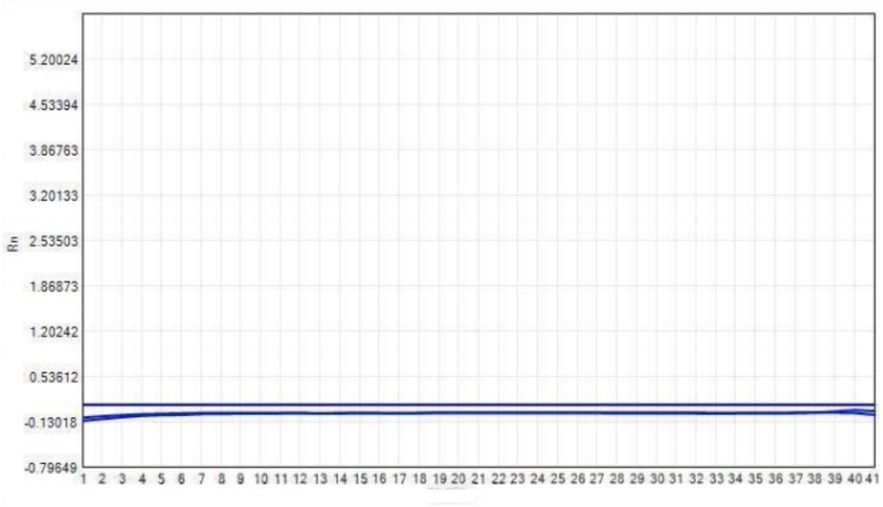

3、 Test Results Summary Table

Test article	Sample Name	Ct	Qualitative findings	Remarks
Positive reference standard	/	21.51 /21.08	Positive result conf-irmed	/
Negative reference material	/	NoCt/NoCt	Negative result confirmed	/
1	MDA-MB-231	NoCt	Negative	/
2	MDA-MB-231	36.17	Negative	/
3	MDA-MB-453	34.41	Negative	/
4	MDA-MB-453	NoCt	Negative	/
5	BT-549	35.68	Negative	/
6	BT-549	35.27	Negative	/
7	MDA-MB-436	NoCt	Negative	
8	MDA-MB-436	NoCt	Negative	

Note: Each sample should be tested in duplicate; if significant differences are observed in the duplicate test results, the sample should be retested.

FUHENG BIOLOGY

Cellular Genetic Quality Assessment Test

Cell Line Authentication Service

STR Genotype Testing Report

Sample Name: clone

Testing Entity: Shanghai Fuheng Biotechnology Co., Ltd.

report date: 2024-06-05

Sample Information

Sample Number:

Customer Sample Number	Company Number
BT-549	240603F

sample size: 1

Sample characteristics: clone

surve llance project: STR

Detection method: Extract genomic DNA from cell lines using Chelex 100 → Perform multiplex PCR using the Si FaSTR 23-plex Kit → Conduct capillary electrophoresis on an ABI 3130 XL Genetic Analyzer → Analyze the data using GeneMapper ID v 3.2 software → Perform data alignment against the EXPASY database.

Detection Result

(1) Basic Inspection Information

Sample Genotype Testing Results

Company Number	Multiple alleles	Matched cell line	Cell bank	EVprice	Matching Instructions
240603F	Not have	BT-549	ExPASy	93.75%	93.75% match

- Multiallelicity refers to the phenomenon of three or more alleles.
- The results of the cellular classification for this test are satisfactory.

(2) Sample Descriptions

240603F: The cell line has been identified as a **human-derived cell line**; its STR profiling data are available in the EXPASY database and show **93.75% similarity to the BT-549 cell line**. This analysis **detected no multiallelic variants or cross-contamination** in this cell line.

Export table

Accession	Name	Nº Markers	Score	Amel	CSF1PO	D2S1338	D3S1358	D5S818	D7S820	D8S1179	D13S317	D16S539	D18S51	D19S433	D21S11	FGA	Penta D	Penta E	TH01	TPOX	vWA
NA	Query	NA	NA		10,12		18	11	9,10	16	11	8	15		32.2	19			9.3	8	15,18
CVCL_1092 *Best*	BT-549	13	93.75%	X	10,12	17	18	11	9,10	14,16	11	8	15	15.2	32.2	19	13	14	9.3	8	15
CVCL_1092 *Worst*	BT-549	13	83.87%	X	10	17	18	11	9	14,16	11	8	15	15.2	28,32.2	19	13	14	9.3	8	15

- **Note:**The test cell lines will be compared against the STR reference data contained in the ExPASy Cell Bank (ExPASy includes approximately 8,701 human cell strains with STR data from databases such as ATCC, DSMZ, JCRB, ECACC, and RIKEN; last updated in May 2024). Cell lines for which no STR data is available will not be eligible for comparison. According to the ATCC Standardization Committee's identification criteria (ANSI/ATCC ASN-0002-2022), a matching degree of EV ≥ 80% indicates that the cell lines are related and may have descended from a common ancestral cell; for matching degrees between 55% and 80%, additional methods must be employed to further identify and verify their relatedness.

(3) Sample Typing Results

Genotyping results for the STR loci and Amelogenin locus in Cell Line 240603F						
Loci	Cell STR profiling data for submission			Cell Bank Cell STR Information		
	Sample cell line: BT-549			Cell Bank Cell Name: BT-549		
	Allele1	Allele2	Allele3	Allele1	Allele2	Allele3
Amelogenin	X					
D3S1358	18			18		
D5S818	11			11		
D2S1338	17					
TPOX	8			8		
CSF1PO	10	12		10	12	
Penta D	13					
TH01	9.3			9.3		
vWA	15	*18*		15		
D7S820	9	10		9	10	
D21S11	32.2			32.2		
Penta E	14					
D10S1248	14					
D8S1179	16			*14*	16	
D1S1656	12	17.3				
D18S51	15			15		
D12S391	18	20				
D6S1043	11					
D19S433	15.2					
D16S539	8			8		
D13S317	11			11		
FGA	19			19		

*Although this table lists the allele data for all loci, the matching algorithm compares only 13 core loci.

Other Notes

(1) Classification Scheme and Site Distribution

Experimental Protocol and Experimental Sites

	Option 1	Option 2	Option 3	Option 4
1	D3S1358	Amelogenin	D10S1248	D19S433
2	D5S818	TH01	D8S1179	D16S539
3	D2S1338	vWA	D1S1656	D13S317
4	TPOX	D7S820	D18S51	FGA
5	CSF1PO	D21S11	D12S391	
6	Penta D	Penta E	D6S1043	

(2) STR Database Comparison

STR typing data comparison is performed against the EXPASY database, which contains STR data for 8,701 human-derived cell lines sourced from databases including ATCC, DSMZ, JCRB, and RIKEN (Cellosaurus, release 49.0; last updated in May 2024). If the target cells are not included in the EXPASY cell bank or if they represent a newly established cell line, the STR typing data comparison cannot be performed; in such cases, users should manually cross-reference the cell typing results with other databases.

Applied Biosystems

240604

GeneMapper ID v3.2

Sample Name	Panel	SQO	SQ
D407	SifaSTR 23 plex_v7.0		

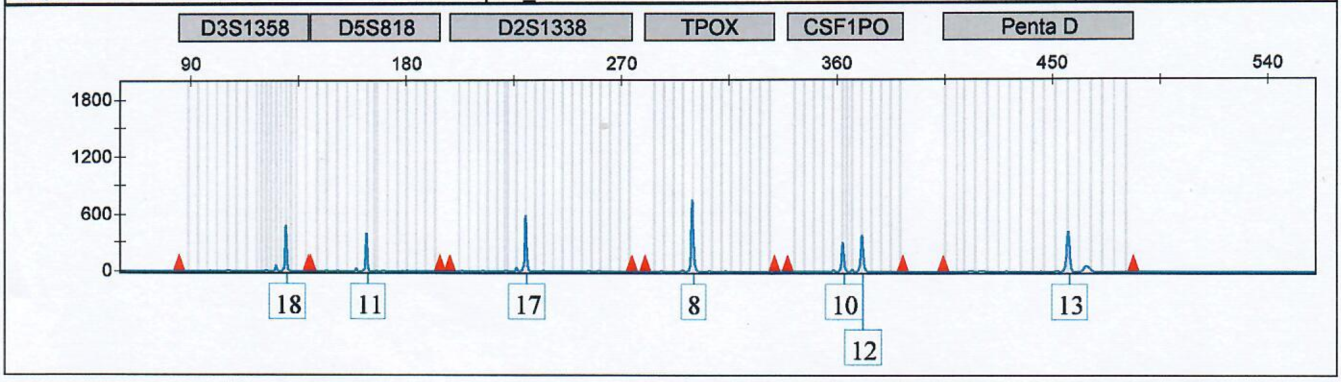

D407	SifaSTR 23 plex_v7.0		

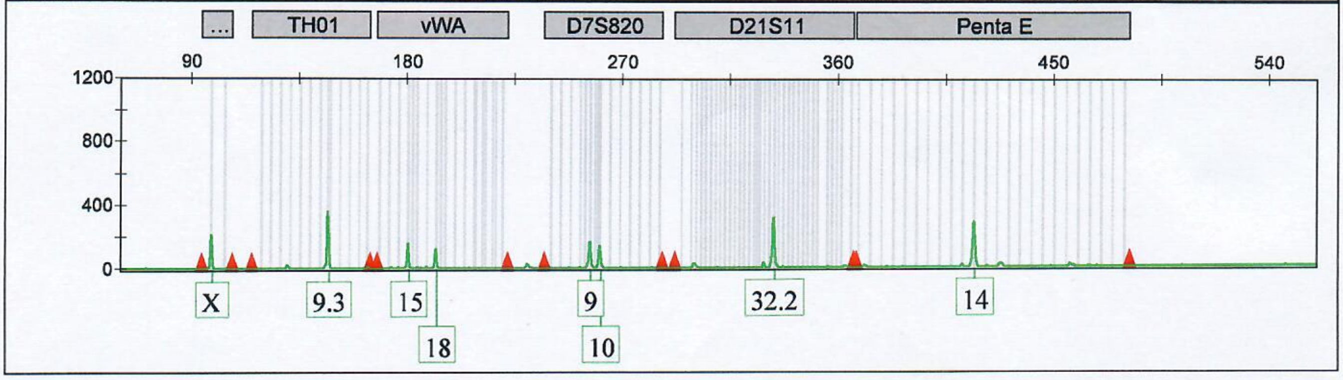

D407	SifaSTR 23 plex_v7.0		

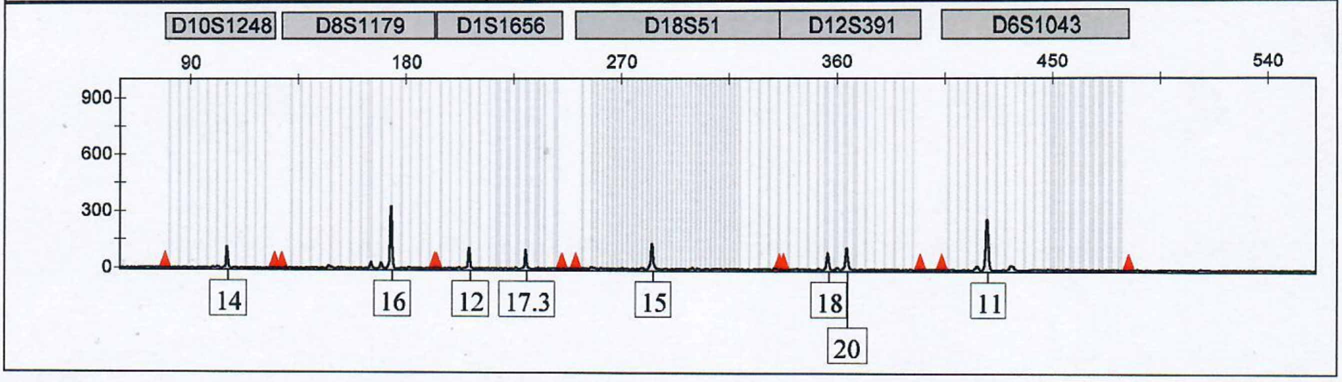

D407	SifaSTR 23 plex_v7.0		

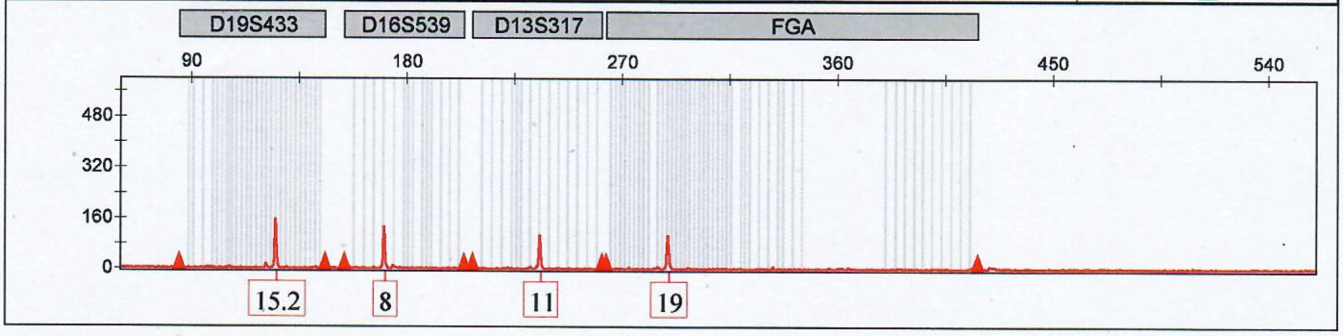

Cellular Genetic Quality Assessment Test

Cell Line Authentication Service

STR Genotype Testing Report

Sample Information

Sample Number:

Sample Number	Company ID
MDA-MB-231	240528I

Sample Size: 1

Sample characteristics: clone

surveillance project: STR

Testing Entity: Shanghai Fuheng Biotechnology Co., Ltd.

Detection method: Extract genomic DNA from cell lines using Chelex 100 → Perform multiplex PCR using the Si FaSTR 23-plex Kit → Conduct capillary electrophoresis on an ABI 3130 XL Genetic Analyzer → Analyze the data using GeneMapper® ID v 3.2 software → Compare the data against the Expasy database.

Detection Result

(1) Basic Inspection Information

Sample Genotype Testing Results

Company ID	Multiple alleles	Matched cell line	Cell bank	EV price	Matching Instructions
240528I	Have	MDA-MB-231	ExPASy	92.68%	92.68% match

- Polymorphism refers to the phenomenon of three or more alleles.
- The results of all cell subtyping analyses in this test were satisfactory.

(2) Sample Descriptions

240528I: The cell line was identified as a **human-derived cell line**; its STR profiling data are available in the EXPASY database and show **92.68% matching with the MDA-MB-231 cell line**. This analysis **detected multiple alleles in this cell line, with no cross-contamination observed**.

Export table

Accession	Name	N° Markers	Score	Amel	CSF1PO	D2S1338	D3S1358	D5S818	D7S820	D8S1179	D13S317	D16S539	D18S51	D19S433	D21S11	FGA	Penta D	Penta E	TH01	TPOX	vWA
NA	Query	NA	NA		12,13		16	12	8,9	13	13	12	11		33.2	22,23			7,9.3	8,9	15,16,18,20
CVCL_0062 *Best*	MDA-MB-231	13	92.68%	X	12,13	20,21	16	12	8,9	13	13	12	11,16	11,14	33.2	22,23	11,14	11	7,9.3	8,9	15,18
CVCL_0062 *Worst*	MDA-MB-231	13	85.71%	X	12,13	20,21	16	12	8,9	13	13	12	11,16	11,14	30,33.2	22,23	11,14	11	7,9.3	8,9	15,19
CVCL_VR36	MDA231-BrM2-831	13	87.18%	X	12,13	20,21	16	12	8	13	13	12	11,16	11,14	33.2	22,23	14	11	7,9.3	8,9	15
CVCL_5998	MDA231-LM2-4175	13	87.18%	X	12,13	20,21	16	12	8	13	13	12	11,16	11,14	33.2	22,23	14	11	7,9.3	8,9	15
CVCL_VR35	MDA231-TGL	13	87.18%	X	12,13	20,21	16	12	8	13	13	12	11,16	11	33.2	22,23	14	11	7,9.3	8,9	15
CVCL_DP48	MDA-BoM-1833	13	85.00%	X	12,13	20,21	16	12	8	13	13	12	11,16	11	30,33.2	22,23			7,9.3	8,9	15

- **Note:**The test cell lines will be compared against the STR reference data contained in the ExPASy Cell Bank (ExPASy includes approximately 8,701 human-derived cell strains with STR data from databases such as ATCC, DSMZ, JCRB, ECACC, and RIKEN; last updated in May 2024). Cell lines for which no STR data is available cannot be compared. According to the identification criteria of the ANSI/ATCC ASN-0002-2022 standardization committee, a matching degree of EV ≥ 80% indicates that the cell lines are related and may have descended from a common ancestral cell; for matching degrees between 55% and 80%, additional methods must be employed to further identify and verify their relatedness.

(3) Sample Genotyping Results

**Genotyping results for the STR loci and the Amelogenin locus in cell line 240528I**						
Loci	Cellular STR profiling data for submission			Cell Bank Cell STR Information		
	Cell line submitted for analysis: MDA-MB-231			Cell bank cell line: MDA-MB-231		
	Allele1	Allele2	Allele3	Allele1	Allele2	Allele3
Amelogenin	X					
D3S1358	16			16		
D5S818	12			12		
D2S1338	20	21				
TPOX	8	9		8	9	
CSF1PO	12	13		12	13	
Penta D	14					
TH01	7	9.3		7	9.3	
vWA	15	*16*	18/*20*	15	18	
D7S820	8	9		8	9	
D21S11	33.2			33.2		
Penta E	11					
D10S1248	16					
D8S1179	13			13		
D1S1656	15	17				
D18S51	11			11	*16*	
D12S391	17	18				
D6S1043	18					
D19S433	11	14				
D16S539	12			12		
D13S317	13			13		
FGA	22	23		22	23	

*Although this table lists allele data for all loci, the matching algorithm compares only 13 core loci.

Other Notes

(1) Classification Scheme and Site Distribution

Experimental Protocol and Experimental Sites

	Solution 1	Option 2	Option 3	Solution 4
1	D3S1358	Amelogenin	D10S1248	D19S433
2	D5S818	TH01	D8S1179	D16S539
3	D2S1338	vWA	D1S1656	D13S317
4	TPOX	D7S820	D18S51	FGA
5	CSF1PO	D21S11	D12S391	
6	Penta D	Penta E	D6S1043	

(2) STR Database Comparison

STR typing data comparison is performed against the EXPASY database, which contains STR data for 8,701 human-derived cell lines sourced from databases including ATCC, DSMZ, JCRB, and RIKEN (Cellosaurus, Release 49.0; last updated in May 2024). If the target cells are not listed in the EXPASY cell bank or if they represent a newly established cell line, the STR typing data comparison cannot be conducted; in such cases, users should perform additional comparisons with other databases based on the cell typing results.

Applied Biosystems

GeneMapper ID v3.2

240528

Sample Name	Panel	SQO	SQ
MDA-MB-231	SifaSTR 23 plex_v7.0		

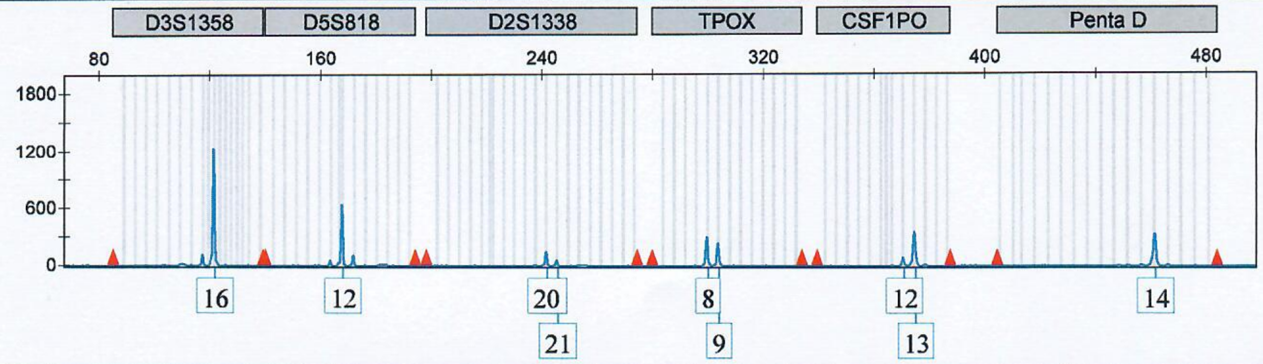

MDA-MB-231	SifaSTR 23 plex_v7.0		

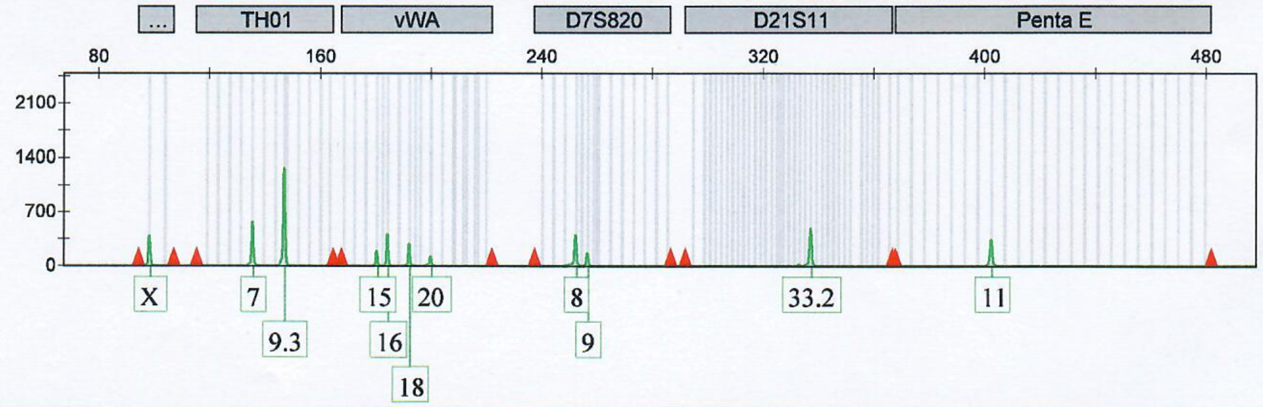

MDA-MB-231	SifaSTR 23 plex_v7.0		

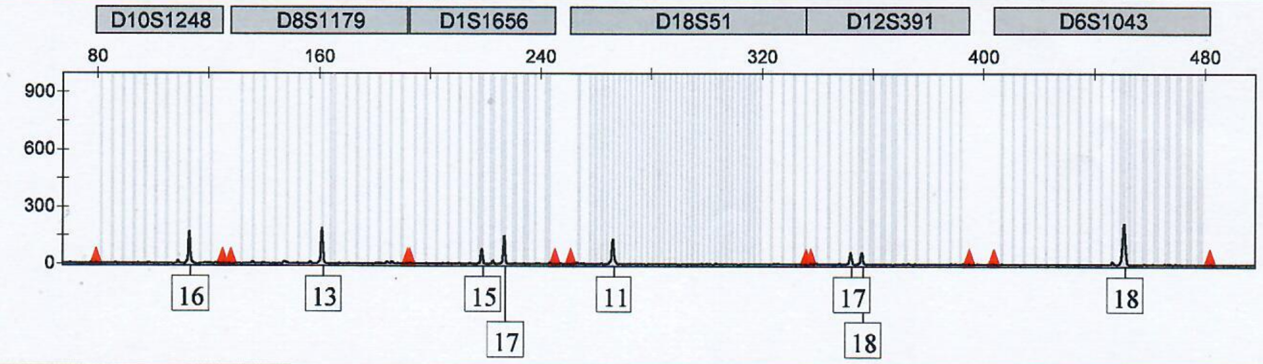

MDA-MB-231	SifaSTR 23 plex_v7.0		

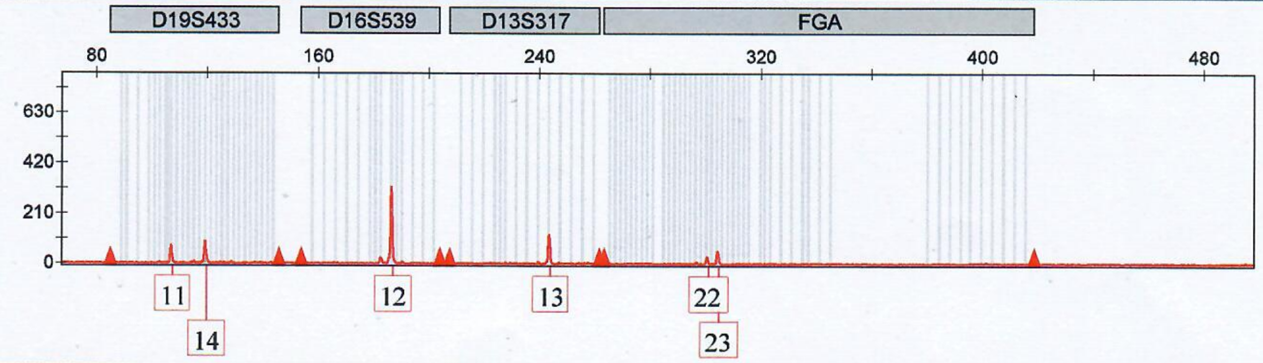

Cellular Genetic Quality Assessment Test

Cell Line Authentication Service

STR Genotype Testing Report

Sample Information

Sample Number:

Sample Number	Company Number
MDA-MB-436	260911P

Sample quantity: 1

Sample characteristics: Cell line

surveillance project:STR

Detection method: Extraction of genomic DNA from cell lines using Chelex 100 → Multiplex PCR performed with the SiFaSTR 23-plex Kit → Capillary electrophoresis conducted on an ABI 3130 XL Genetic Analyzer → Data analysis performed using GeneMapper® ID v3.2 software → Data comparison performed against the ExPASy database.

detection result

(一) Check basic information

Sample genotype testing results

Company Number	multiple alleles	Matched cell line	cell bank	EVprice	Matching Instructions
260911P	not have	MDA-MB-436	ExPASy	100%	Exact match

- Polymorphism refers to the phenomenon involving three or more alleles.
- The results of the current cellular classification test are satisfactory.

(二) Sample Description

260911P: The cell line identification confirms it as a human-derived cell line; its STR profiling data are available in the ExPASy database and show 100% matching with the MDA-MB-436 cell line. No multiallelic variants were detected in this cell line, and no cross-contamination was observed.

Export table

Accession	Name	N° Markers	Score	Ame	CSF1PO	D2S1338	D3S1358	D5S818	D7S820	D8S1179	D13S317	D16S539	D18S51	D19S433	D21S11	FGA	Penta D	Penta E	TH01	TPOX	vWA
NA	Query	NA	NA		12		18	13	10	10,14	10	9,11	12		30,31.2	24			9.3	8	14,20
CVCL_0623 *Best*	MDA-MB-436	13	100.00%	X	12	23	18	13	10	10,14	10	9,11	12	13	30,31.2	24	9	10,12	9.3	8	14,20
CVCL_0623 *Worst*	MDA-MB-436	13	94.12%	X	12	23	18	13	10	10,14	10	9	12	13	30,31.2	21,24	9	10,12	9.3	8	14,20

- Note: The test cell lines will be compared against the STR reference data recorded in the ExPASy Cell Bank (ExPASy contains approximately 9,807 human-derived cell strains' STR data from databases including ATCC, DSMZ, JCRB, ECACC, and RIKEN; last updated in June 2026). Cell lines for which no STR data is available will not be eligible for comparison. According to the identification criteria established by the ANSI/ATCC ASN-0002-2022 standardization committee, a matching degree of EV ≥ 80% indicates that the cell lines are related and may have descended from a common ancestral cell; for matching degrees between 55% and 80%, additional methods must be employed to further verify their relatedness.

(三) Sample genotyping results

<table>
<tr><th colspan="3">Genotyping results for STR loci and Amelogenin locus in cell line 260911P</th></tr>
<tr><td rowspan="2">Loci</td><td>Cellular STR profiling data for submission</td><td>Cell Bank STR Information</td></tr>
<tr><td>Cell line submitted for analysis: MDA-MB-436</td><td>Cell Bank Cell Name: MDA-MB-436</td></tr>
</table>

	Allele1	Allele2	Allele3	Allele1	Allele2	Allele3
Amelogenin	X					
D3S1358	18			18		
D5S818	13			13		
D2S1338	23					
TPOX	8			8		
CSF1PO	12			12		
Penta D	9					
TH01	9.3			9.3		
vWA	14	20		14	20	
D7S820	10			10		
D21S11	30	31.2		30	31.2	
Penta E	10	12				
D10S1248	13	14				
D8S1179	10	14		10	14	
D1S1656	17.3	19.3				
D18S51	12			12		
D12S391	20					
D6S1043	11					
D19S433	13					
D16S539	9	11		9	11	
D13S317	10			10		
FGA	24			24		

Although this table lists allele data for all loci, the matching algorithm compares only the 13 core loci.

Other Notes

(一) Classification scheme and locus distribution

Experimental Protocol and Experimental Sites

	Option 1	Option 2	Option 3	Option 4

1	D3S1358	Amelogenin	D10S1248	D19S433
2	D5S818	TH01	D8S1179	D16S539
3	D2S1338	vWA	D1S1656	D13S317
4	TPOX	D7S820	D18S51	FGA
5	CSF1PO	D21S11	D12S391	
6	Penta D	Penta E	D6S1043	

(二) STR database alignment

STR typing data comparison is performed against the ExPASy database, which contains STR data for 9,807 human-derived cell lines obtained from databases including ATCC, DSMZ, JCRB, and RIKEN (Cellosaurus release 56.0, last updated in June 2026). If the target cells are not included in the ExPASy cell bank or if they represent a newly established cell line, the STR typing data comparison cannot be performed; in such cases, users should manually compare the cell typing results with other databases.

Applied Biosystems

GeneMapper ID v3.2

260911

Sample Name	Panel	SQO	SQ
MDA-MB-436	SifaSTR 23 plex_v7.0		

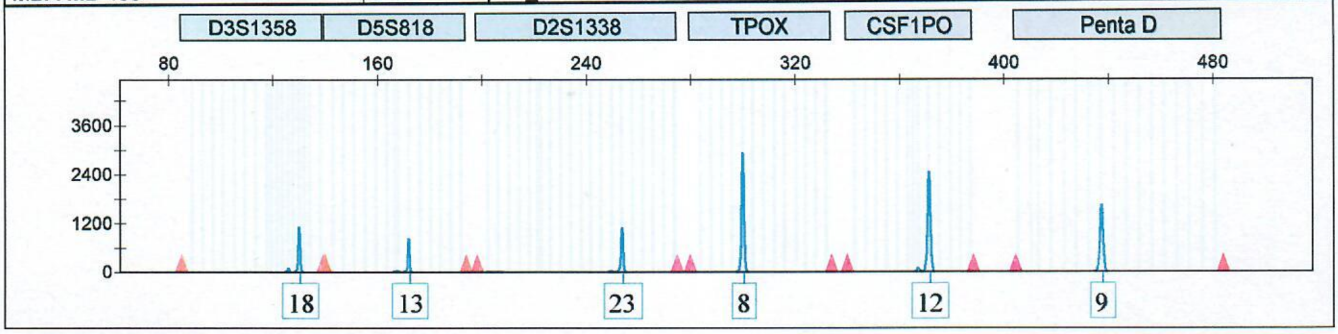

MDA-MB-436	SifaSTR 23 plex_v7.0		

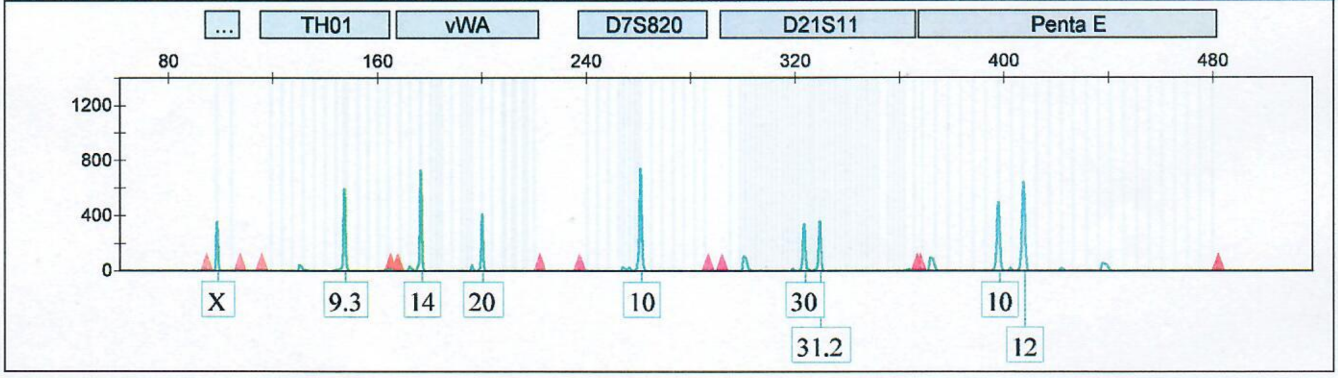

MDA-MB-436	SifaSTR 23 plex_v7.0		

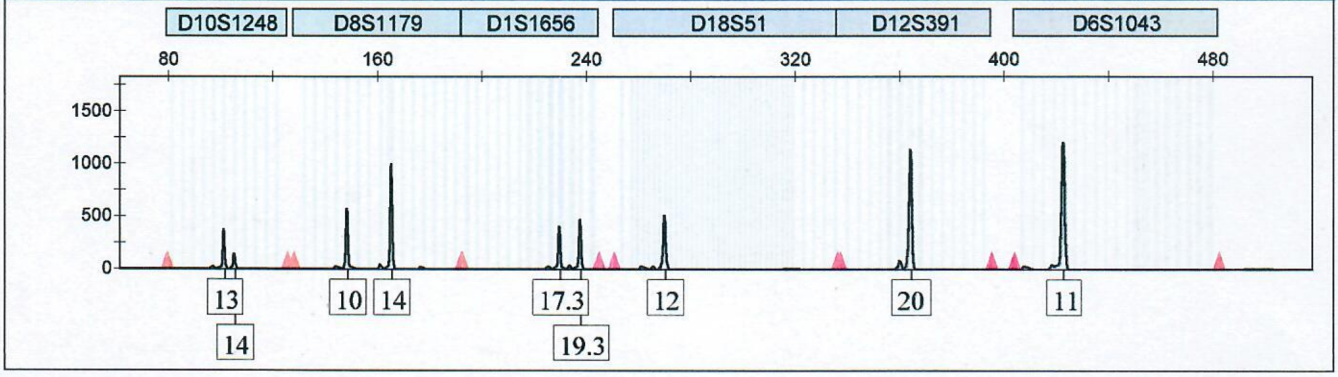

MDA-MB-436	SifaSTR 23 plex_v7.0		

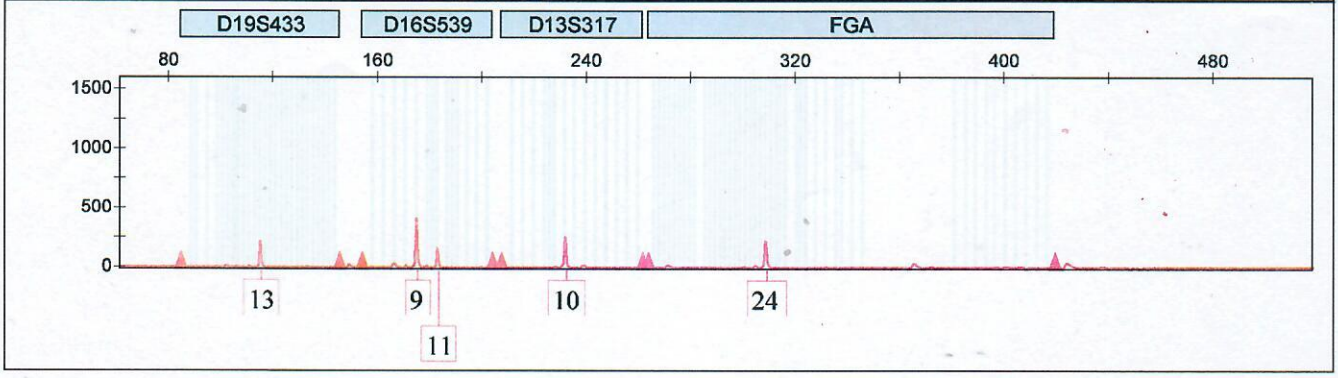

Cellular Genetic Quality Assessment Test

Cell Line Authentication Service

STR Genotype Testing Report

Sample Information

Sample Number:

Sample Number	Company Number
MDA-MB-453	260911O

Sample quantity: 1

Sample characteristics: Cell line

surveillance project:STR

Detection method: Extraction of genomic DNA from cell lines using Chelex 100 → Multiplex PCR performed with the SiFaSTR 23-plex Kit → Capillary electrophoresis conducted on an ABI 3130 XL Genetic Analyzer → Data analysis performed using GeneMapper® ID v3.2 software → Data comparison performed against the ExPASy database.

detection result

(一) Check basic information

Sample genotype testing results

Company Number	multiple alleles	Matched cell line	cell bank	EVprice	Matching Instructions
260911O	not have	MDA-MB-453	ExPASy	100%	Exact match

- Polymorphism refers to the phenomenon involving three or more alleles.
- The results of the current cellular classification test are satisfactory.

(二) Sample Description

260911O: The identification results confirm that this cell line is a human-derived cell line; its STR profiling data are available in the ExPASy database and show 100% matching with the MDA-MB-453 cell line. No multiallelic variants were detected in this cell line during the current analysis, and no cross-contamination was observed.

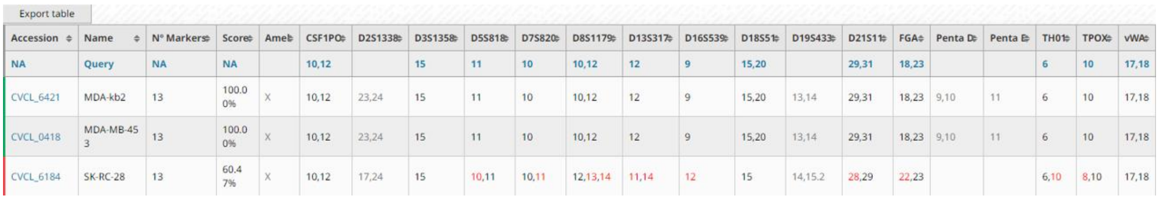

Export table

Accession	Name	N° Markers	Score	Amel	CSF1PO	D2S1338	D3S1358	D5S818	D7S820	D8S1179	D13S317	D16S539	D18S51	D19S433	D21S11	FGA	Penta D	Penta E	TH01	TPOX	vWA
NA	Query	NA	NA		10,12		15	11	10	10,12	12	9	15,20		29,31	18,23			6	10	17,18
CVCL_6421	MDA-kb2	13	100.00%	X	10,12	23,24	15	11	10	10,12	12	9	15,20	13,14	29,31	18,23	9,10	11	6	10	17,18
CVCL_0418	MDA-MB-453	13	100.00%	X	10,12	23,24	15	11	10	10,12	12	9	15,20	13,14	29,31	18,23	9,10	11	6	10	17,18
CVCL_6184	SK-RC-28	13	60.47%	X	10,12	17,24	15	10,11	10,11	12,13,14	11,14	12	15	14,15.2	28,29	22,23			6,10	8,10	17,18

- Note: The test cell lines will be compared against the STR reference data recorded in the ExPASy Cell Bank (ExPASy contains approximately 9,807 human-derived cell strains' STR data from databases including ATCC, DSMZ, JCRB, ECACC, and RIKEN; last updated in June 2026). Cell lines for which no STR data is available will not be eligible for comparison. According to the identification criteria established by the ANSI/ATCC ASN-0002-2022 standardization committee, a matching degree of EV ≥ 80% indicates that the cell lines are related and may have descended from a common ancestral cell; for matching degrees between 55% and 80%, additional methods must be employed to further verify their relatedness.

(三) Sample genotyping results

Genotyping results for STR loci and Amelogenin locus in cell line 260911O		
Loci	Cellular STR profiling data for submission	Cell Bank STR Information
	Cell line submitted for analysis: MDA-MB-453	Cell Bank Cell Name: MDA-MB-453

	Allele1	Allele2	Allele3	Allele1	Allele2	Allele3
Amelogenin	X					
D3S1358	15			15		
D5S818	11			11		
D2S1338	23	24				
TPOX	10			10		
CSF1PO	10	12		10	12	
Penta D	9	10				
TH01	6			6		
vWA	17	18		17	18	
D7S820	10			10		
D21S11	29	31		29	31	
Penta E	11					
D10S1248	14	15				
D8S1179	10	12		10	12	
D1S1656	16.3	17.3				
D18S51	15	20		15	20	
D12S391	18	21				
D6S1043	11	18				
D19S433	13	14				
D16S539	9			9		
D13S317	12			12		
FGA	18	23		18	23	

Although this table lists allele data for all loci, the matching algorithm compares only the 13 core loci.

Other Notes

(一) Classification scheme and locus distribution

Experimental Protocol and Experimental Sites

	Option 1	Option 2	Option 3	Option 4
1	D3S1358	Amelogenin	D10S1248	D19S433
2	D5S818	TH01	D8S1179	D16S539

3	D2S1338	vWA	D1S1656	D13S317
4	TPOX	D7S820	D18S51	FGA
5	CSF1PO	D21S11	D12S391	
6	Penta D	Penta E	D6S1043	

（二） STR database alignment

STR typing data comparison is performed against the ExPASy database, which contains STR data for 9,807 human-derived cell lines obtained from databases including ATCC, DSMZ, JCRB, and RIKEN (Cellosaurus release 56.0, last updated in June 2026). If the target cells are not included in the ExPASy cell bank or if they represent a newly established cell line, the STR typing data comparison cannot be performed; in such cases, users should manually compare the cell typing results with other databases.

Applied Biosystems

GeneMapper ID v3.2

260911

Sample Name	Panel	SQO	SQ
MDA-MB-453	SifaSTR 23 plex_v7.0		

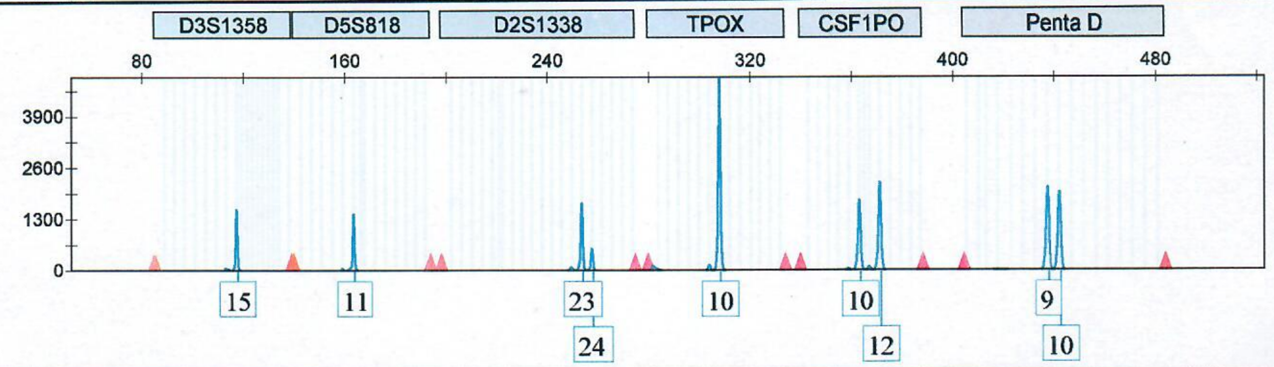

MDA-MB-453	SifaSTR 23 plex_v7.0		

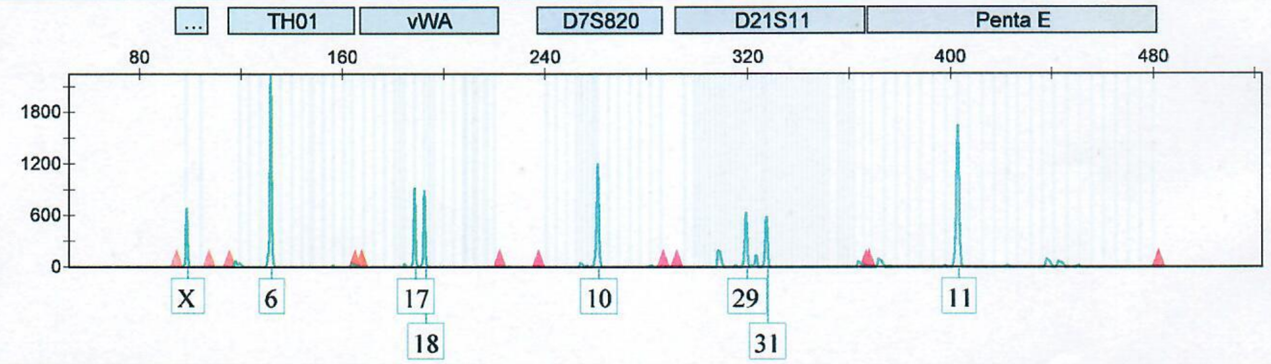

MDA-MB-453	SifaSTR 23 plex_v7.0		

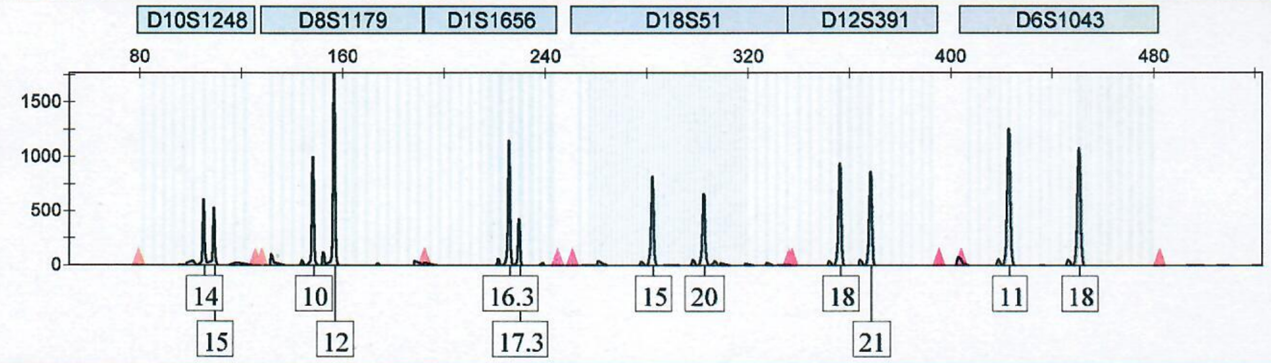

MDA-MB-453	SifaSTR 23 plex_v7.0		

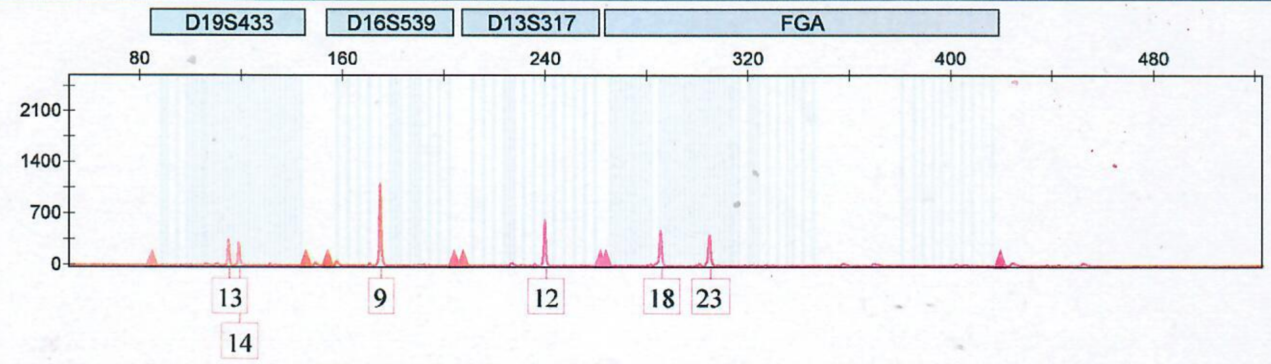

Supplement: Supplementary file 1 [file cancers-18-03156-s001.zip › STR authentication and mycoplasma testing reports for cell lines.pdf]
